# Multilocus Sequence Analysis for Assessment of Phylogenetic Diversity and Biogeography in *Thalassospira* Bacteria from Diverse Marine Environments

**DOI:** 10.1371/journal.pone.0106353

**Published:** 2014-09-08

**Authors:** Qiliang Lai, Yang Liu, Jun Yuan, Juan Du, Liping Wang, Fengqin Sun, Zongze Shao

**Affiliations:** 1 State Key Laboratory Breeding Base of Marine Genetic Resources, Xiamen, China; 2 Key Laboratory of Marine Genetic Resources, Third Institute of Oceanography, SOA, Xiamen, China; 3 Key Laboratory of Marine Genetic Resources of Fujian Province, Xiamen, China; 4 Collaborative Innovation Center of Deep Sea Biology, Xiamen, China; 5 Fujian Collaborative Innovation Center for Exploitation and Utilization of Marine Biological Resources, Xiamen, China; The University of Hong Kong, Hong Kong

## Abstract

*Thalassospira* bacteria are widespread and have been isolated from various marine environments. Less is known about their genetic diversity and biogeography, as well as their role in marine environments, many of them cannot be discriminated merely using the 16S rRNA gene. To address these issues, in this report, the phylogenetic analysis of 58 strains from seawater and deep sea sediments were carried out using the multilocus sequence analysis (MLSA) based on *acsA*, *aroE*, *gyrB*, *mutL*, *rpoD* and *trpB* genes, and the DNA-DNA hybridization (DDH) and average nucleotide identity (ANI) based on genome sequences. The MLSA analysis demonstrated that the 58 strains were clearly separated into 15 lineages, corresponding to seven validly described species and eight potential novel species. The DDH and ANI values further confirmed the validity of the MLSA analysis and eight potential novel species. The MLSA interspecies gap of the genus *Thalassospira* was determined to be 96.16–97.12% sequence identity on the basis of the combined analyses of the DDH and MLSA, while the ANIm interspecies gap was 95.76–97.20% based on the in silico DDH analysis. Meanwhile, phylogenetic analyses showed that the *Thalassospira* bacteria exhibited distribution pattern to a certain degree according to geographic regions. Moreover, they clustered together according to the habitats depth. For short, the phylogenetic analyses and biogeography of the *Thalassospira* bacteria were systematically investigated for the first time. These results will be helpful to explore further their ecological role and adaptive evolution in marine environments.

## Introduction

*Thalassospira* are a genus consisting of Gram-negative, motile, vibrio- or spiral-shaped, halotolerant and chemoheterotrophic bacteria belonging to the family *Rhodospirillaceae* within the class *Alphaproteobacteria* and was created by López-López *et al* in 2002 [1]. In the following years, more and more isolates of *Thalassospira* were obtained from various marine environments and identified using the polyphasic taxonomy. Currently, the *Thalassospira* genus has accommodated eight species: *T. lucentensis* [1], *T. profundimaris* [2], *T. xiamenensis* [2], *T. tepidiphila* [3], *T. xianhensis* [4], *T. alkalitolerans* and *T. mesophila* [5], *T. povalilytica* [6], except a non-validly published species named *T. permensis* [7].

We found that bacteria of *Thalassospira* were widespread in the marine environments and occupied a variety of ecological niches, such as surface and deep seawater, deep sediment, halobios etc, covering the Pacific Ocean, the Atlantic Ocean, the Indian Ocean and even the Arctic Ocean [8]–[11]. They aroused extensive attention because of their potential in eliminating marine oil pollution, especially in polycyclic aromatic hydrocarbons (PAHs) degradation [3], [4], [7], [12]. Recently, *Thalassospira* were found to closely related with the phosphorus cycling in marine environments, especially in the oligotrophic open ocean environments [13], [14]. Additionally, some strains of this genus can produce thalassospiramides [15], [16], beta-galactosidase [17] and biosurfactants [18]. Accumulation of these bacteria with different functions and originations urges analysis on their diversity, evolution and geographic distribution.

In recent years, a large number of bacteria within the genus *Thalassospira* were isolated from various marine samples enriched with different hydrocarbons, including hexadecane, BTEX (benzene, toluene, ethylbenzene and xylene), and crude oil in our laboratory. Nevertheless, most of the *Thalassospira* bacteria shared relatively high identities to the 16S rRNA gene (>98%) with each other, with only one exception of *T. lucentensis* DSM 14000^T^, thus they could not be classified based upon the 16S rRNA gene. Therefore, an effective method is imperative to discriminate these closely related strains.

Since 2005, MLSA has rapidly become a powerful tool and has been performed frequently for the taxonomy and phylogenetic analysis of the closely related strains [19]–[21]. In general, the several housekeeping genes are widely applied in MLSA schemes, such as *acsA*, *gyrB*, *mutL*, *rpoD*, etc [22]–[25], because they encode core metabolic enzymes and own by all members of the organisms. Furthermore, the concatenated housekeeping genes could overcome conflicting signals from horizontal gene transfer and recombination. In addition, DNA fingerprinting [26], [27], DNA-DNA hybridization (DDH) [21], [23] and average nucleotide identity (ANI) [28], [29] are dependable methods for identification of the closely related strains.

In this study, we employed MLSA using six housekeeping genes, *acsA*, *aroE*, *gyrB*, *mutL*, *rpoD* and *trpB*, combined with the 16S rRNA gene, DDH and ANI values from draft genome sequences, to explore the diversity, and geographic distribution of the *Thalassospira* bacteria originating from diverse marine environments.

## Materials and Methods

### Ethics Statement

The bacterial strains in this study were isolated from the various areas beyond the international sea area or the exclusive economic zone of China. The type strains were obtained from the public culture collections. So no specific permissions were required for these collections. Moreover, the sample did not involve endangered or protected species.

### Bacterial strains

A total of 58 *Thalassospira* strains were analyzed in this study. Specifically, 52 *Thalassospira* strains were isolated in our laboratory and deposited at the Marine Culture Collection of China (MCCC), including two type strains, *T. xiamenensis* M-5^T^ and *T. profundimaris* WPO0211^T^. *T. tepidiphila* 1-1B^T^ was generous gift from Dr. Kazuya Watanabe [3]; *T. lucentensis* DSM 14000^T^, *T. xianhensis* CGMCC 1.6849^T^, *T. alkalitolerans* JCM 18968^T^, *T. mesophila* JCM 18969^T^ and *T. permensis* NBRC 106175^T^ (non-valid) were purchased from DSMZ, CGMCC, JCM and NBRC, respectively. The detail information of all strains was presented in Table 1. They were isolated from 32 different locations across the Pacific Ocean, the Atlantic Ocean, the Indian Ocean, the Yellow Sea, the East China Sea and the Mediterranean Sea. The sampling sites were shown in Figure S1. At the time of this study, type strain *T. povalilytica* Zumi 95^T^ was not reported and therefore not included in our study.

**Table 1:** The 58 *Thalassospira* bacteria in this study.

MCCC NO.	Original No.	Closest type strain of 16S rRNA gene (Identity %)	Geographic origin	Source	Depth (m)
1A00207	WP0211^T^	*T. profundimaris*	The Pacific Ocean	Sediment	4,480
1A00209	M-5^T^	*T. xiamenensis*	The Taiwan strait	Superficial seawater	0
1A00350	R8-17	*T. profundimaris* (99.7)	The Indian Ocean	Deep seawater	668
1A00370	R8-8	*T. profundimaris* (99.4)	The Indian Ocean	Deep seawater	668
1A00383	QMT2^T^	*T. lucentensis*	The Mediterranean Sea	Upper seawater	100
1A00385	R4-5	*T. profundimaris* (99.4)	The Indian Ocean	Deep seawater	2,268
1A00624	SMB34^T^	*T. permensis*	Berezniki, Perm region, Russia	Soil	0
1A00753	MBE#61^T^	*T. alkalitolerans*	The coastal area, Japan	Sunken bamboo	0
1A00756	MBE#74^T^	*T. mesophila*	The coastal area, Japan	Sunken bamboo	0
1A01013	W3-1	*T. xiamenensis* (99.4)	The Pacific Ocean	Sediment	2,682
1A01017	DBT-2	*T. xiamenensis* (99.4)	The Pacific Ocean	Sediment	2,682
1A01041	PTG4-18	*T. xiamenensis* (99.5)	The Indian Ocean	Sediment	2,946
1A01051	MARC2PI	*T. xiamenensis* (99.5)	The Atlantic Ocean	Sediment	3,542
1A01057	MARC4CW	*T. profundimaris* (99.7)	The Atlantic Ocean	Sediment	3,962
1A01072	MARC2COD	*T. xiamenensis* (99.5)	The Atlantic Ocean	Sediment	3,542
1A01103	PB8B	*T. profundimaris* (99.6)	The Indian Ocean	Deep seawater	1,800
1A01109	PB9B	*T. profundimaris* (99.4)	The Indian Ocean	Deep seawater	1,800
1A01140	MARMC3G	*T. xiamenensis* (99.5)	The Atlantic Ocean	Sediment	4,046
1A01148	MARC2CO7	*T. profundimaris* (99.7)	The Atlantic Ocean	Sediment	3,542
1A01166	35	*T. profundimaris* (99.4)	The Indian Ocean	Sediment	2851
1A01167	78	*T. profundimaris* (99.4)	The Indian Ocean	Sediment	2,851
1A01172	MARC2PPNC	*T. profundimaris* (99.4)	The Atlantic Ocean	Sediment	3,542
1A01275	MC2-14	*T. profundimaris* (99.7)	The Atlantic Ocean	Deep seawate	3,542
1A01288	S31-2-1	*T. profundimaris* (98.3)	The Indian Ocean	Superficial seawater	0
1A01300	S27-11	*T. xiamenensis* (99.5)	The Indian Ocean	Superficial seawater	0
1A01318	S25-3-2	*T. profundimaris* (98.3)	The Indian Ocean	Superficial seawater	0
1A01330	S29-3-A	*T. xiamenensis* (99.5)	The Indian Ocean	Superficial seawater	0
1A01423	S25-4	*T. profundimaris* (98.3)	The Indian Ocean	Superficial seawater	0
1A01448	S31-7	*T. xiamenensis* (99.5)	The Indian Ocean	Superficial seawater	0
1A01449	S31-6	*T. profundimaris* (98.3)	The Indian Ocean	Superficial seawater	0
1A02030	PR54-5	*T. profundimaris* (99.5)	The Indian Ocean	Deep seawater	4,146
1A02031	2CR55-14	*T. profundimaris* (99.5)	The Indian Ocean	Deep seawater	3,946
1A02039	PR57-5	*T. profundimaris* (99.7)	The Indian Ocean	Deep seawater	3,546
1A02040	PR57-2	*T. profundimaris* (99.7)	The Indian Ocean	Deep seawater	3,546
1A02041	2CR55-15	*T. profundimaris* (99.6)	The Indian Ocean	Deep seawater	3,946
1A02042	2CR-54-5	*T. profundimaris* (99.6)	The Indian Ocean	Deep seawater	4,146
1A02059	NIC1013S-2	*T. profundimaris* (99.4)	The Indian Ocean	Deep seawater	2,455
1A02060	RC911-4	*T. profundimaris* (99.4)	The Indian Ocean	Deep seawater	668
1A02093	PC99-15	*T. profundimaris* (99.7)	The Indian Ocean	Deep seawater	1,068
1A02094	MC2-9	*T. xiamenensis* (99.5)	The Atlantic Ocean	Overlaid seawater	3,542
1A02096	PC92-18	*T. profundimaris* (99.7)	The Indian Ocean	Deep seawater	2,468
1A02616	P-4^T^	*T. xianhensis*	Xianhe town, China	Soil	0
1A02753	IB2	*T. xiamenensis* (99.4)	Yellow Sea, China	Upper seawater	10
1A02758	IB13	*T. xiamenensis* (99.2)	Yellow Sea, China	Upper seawater	10
1A02767	ID7	*T. xiamenensis* (99.2)	Yellow Sea, China	Upper seawater	5
1A02785	IH1	*T. xiamenensis* (99.2)	Yellow Sea, China	Upper seawater	5
1A02803	IK1	*T. profundimaris* (100)	Yellow Sea, China	Upper seawater	40
1A02843	IP8	*T. xiamenensis* (99.2)	Yellow Sea, China	Upper seawater	30
1A02866	IU14	*T. xiamenensis* (99.2)	Yellow Sea, China	Upper seawater	30
1A02873	IV17	*T. xiamenensis* (99.2)	Yellow Sea, China	Upper seawater	40
1A02878	IX2	*T. xiamenensis* (99.4)	Yellow Sea, China	Upper seawater	50
1A02898	JB7	*T. profundimaris* (100)	Yellow Sea, China	Upper seawater	2.5
1A02921	JG3	*T. xiamenensis* (99.4)	East China Sea	Upper seawater	30
1A02935	JK1	*T. xiamenensis* (99.2)	East China Sea	Upper seawater	30
1A03005	L6	*T. xiamenensis* (99.5)	The Atlantic Ocean	Sediment	3,962
1A03052	AS-I2-11	*T. xiamenensis* (99.5)	The Indian Ocean	Sediment	1,420
1A03093	AS-M6-11	*T. xiamenensis* (99.5)	The Atlantic Ocean	Sediment	2,987
1A03514	1-1B^T^	*T. tepidiphila*	Kamaishi Bay, Japan	Upper seawater	15

### Cultivation and DNA preparation

All strains were incubated on 216 L agar medium (CH_3_COONa 1.0 g, tryptone 10.0 g, yeast extract 2.0 g, sodium citrate 0.5 g, NH_4_NO_3_ 0.2 g, agar 15 g, 1 L sea water, pH 7.5) [30] at 28°C for 48 h. Genomic DNA was extracted using the SBS extraction kit (SBS Genetech Co., Ltd. in Shanghai, China) following the manufacturer's instructions.

### PCR amplification and sequencing of 16S rRNA and six housekeeping genes

The 16S rRNA gene was amplified using universal primers 27F and 1492R, while six housekeeping genes were amplified, respectively, with specific primers designed in light of the genome sequences of the six type strains using the Primer Premier 5.0 (Table S1). These genes were amplified under nearly identical conditions. The 50 µL reaction mixtures contained 37 µL of double distilled water, 5 µL of 10×Ex Taq buffer (Mg^2+^ Plus), 4 µL of dNTP mixture (10 mM), 1 µL of each primer (20 µM), 1 µL of genomic DNA template (10–30 ng/μL), 1 µL of Ex Taq™ DNA polymerase (TaKaRa, 5 U/μL). The reaction mixture was subjected to the following parameters in a Biometra T-Professional thermocycler (Biometra; Goettingen, Germany): initial denaturation at 94°C for 5 min; then 30 cycles of 30 s of denaturation at 94°C, 30 s of annealing at 55°C, and 1.0–1.5 min of extension at 72°C; and a final extension at 72°C for 10 min. More detail information was listed in Table S1. After amplification, these PCR amplicons were separated by electrophoresis on a 1% agarose gel and then purified using the PCR purification kit (Axygen Scientific, Inc., USA) according to the manufacturer's instructions. Finally, the purified amplicons were sequenced with the ABI3730xl platform (Shanghai Majorbio Bio- Pharm Technology Co., Ltd., China) using the corresponding sequencing primers (Table S1).

The sequences of the 16S rRNA gene and six housekeeping genes were assembled and modified using DNAMAN version 5.0. All sequences were submitted to the GenBank database. The accession numbers were assigned and listed in Table S2.

### Analysis of nucleotide diversity

The determined sequences of the 16S rRNA gene and six housekeeping genes were compared to the National Center of Biotechnology Information (NCBI) database using BLAST. For each housekeeping gene, different alleles were assigned successively different numbers, and a unique combination of 6 allele numbers (allelic profile) unambiguously was defined the sequence type (ST) of a bacterium. When several strains shared the same allelic profiles, they unquestionably possessed the same ST. A total of allelic profiles were utilized for subsequent analysis. Meantime, the genetic distances and sequence similarities of the single gene were calculated using Kimura's 2-parameter model [31] with the MEGA version 5.0 [32]. The numbers of polymorphic sites and the mean G+C (guanine-cytosine) content in each gene were obtained by the MEGA version 5.0. The nucleotide diversity (π) per site for each gene was performed using the data analysis in molecular biology and evolution (DAMBE) version 5.0 [33]. In addition, the selective pressure on six housekeeping genes were evaluated with the ratios of *K*a/*K*s (*K*a: the number of non-synonymous substitutions per non-synonymous site, *K*s: the number of synonymous substitutions per synonymous site) using the MEGA version 5.0.

### Population genetic analyses

The assessment of substitution saturation for each gene was determined using the DAMBE version 5.0. In order to measure the possibility of reticulate evolution, split decomposition analysis was performed for all single gene and the concatenated genes using the neighbor-net algorithm in SplitsTree version 4.0 by default settings [34]. The possible existence of the recombination in all genes was estimated using the pairwise homoplasy index (PHI) test as implemented in SplitsTree version 4.0. Meantime, the level of Linkage disequilibrium, as measured by the standardized index of association (*IS A*), were investigated using the LInkage ANalysis (LIAN) version 3.6 (http://guanine.evolbio.mpg.de/cgi-bin/lian/lian.cgi.pl/query) [35]. In addition, radiation times were estimated with each other based on the rate of synonymous substitution for single housekeeping genes and the concatenated housekeeping genes. These rates were normalized with the known radiation time between *Escherichia coli* and *Salmonella enterica* (ca. 1 million years) [36].

### Phylogenetic analysis based on the 16S rRNA gene, individual housekeeping gene, the six concatenated housekeeping genes and DDH

The phylogenetic analysis was performed using MEGA version 5.0. The sequences of the 16S rRNA gene and six housekeeping genes were aligned using ClustalW option implemented in MEGA. A series of phylogenetic trees based on the 16S rRNA gene and single housekeeping gene were constructed respectively using the neighbour-joining algorithm by Kimura's 2-parameter model with the MEGA version 5.0. A phylogenetic tree based on the concatenated gene (in the following order: *acsA*, *aroE*, *gyrB*, *mutL*, *rpoD* and *trpB*) was also constructed. *Rhodospirillum rubrum* ATCC 11170^T^ was used as the outgroup in all phylogenetic analysis. Bootstrap confidence analysis was carried out with 1000 replications for evaluating the robustness of the tree topologies. Additionally, phylogenies from distance matrix by neighbour-joining method the clustering tree based on the DDH was constructed with the web server (http://genome.csdb.cn/cgi-bin/emboss/fneighbor).

### Correlation analysis between similarities of the MLSA, DDH and ANI

The genome sequences of sixteen representative *Thalassospira* strains selected based on the phylogenetic analysis were determined by Shanghai Majorbio Bio-pharm Technology Co., Ltd. (Shanghai, China), using Solexa paired-end (500 bp library) sequencing technology. A total of 500 M bp clean data for each strain was generated to reach about 100-fold depth of coverage with an Illumina/Solexa Genome Analyzer IIx (Illumina, SanDiego, CA). The clean data was assembled by SOAPdenovo2 [37]. Two of them (*T. profundimaris* WP0211^T^ and *T. xiamenensis* M-5^T^) had been reported by our lab [38], [39]. DDH values were estimated using the genome-to-genome distance calculator website service (GGDC 2.0) [40], [41]. ANI values of these strains were calculated using the software JSpecies (V1.2.1) [29]. Correlation analysis between similarities of the MLSA and DDH was simulated using the R version 3.0.1.

## Results

### Individual gene analyses

The sequences of the 16S RNA gene and the six housekeeping genes of 58 strains were determined. The characteristics of gene(s) were listed in Table 2.

**Table 2:** Characteristics of the 16S rRNA gene, single housekeeping gene and the concatenated genes from 58 strains.

Locus	Length (bp)	No. of Alleles	No. (%) of Polymorphic sites	π	Mean G+C content (mol %)	K_2_P distance range	K_2_P distance mean	*K*a/*K*s
16S rDNA	1,444–1,449	20	64 (4.42–4.43)	0.012	55.1	0.000–0.027	0.009	NA
*acsA*	858	24	371 (43.2)	0.180	53.4	0.000–0.247	0.170	0.035
*aroE*	663	25	354 (53.4)	0.192	54.7	0.000–0.345	0.190	0.099
*gyrB*	918	25	368 (40.1)	0.135	56.1	0.000–0.218	0.124	0.052
*mutL*	726	25	298 (40.0)	0.148	60.8	0.000–0.215	0.140	0.039
*rpoD*	510	25	179 (35.1)	0.119	53.1	0.000–0.192	0.111	0.060
*trpB*	738	28	280 (38.0)	0.140	57.2	0.000–0.221	0.134	0.049
MLSA	4,413	31	1,850 (41.9)	0.148	56.0	0.000–0.220	0.138	NA

Nucleotide diversity (π): the average number of nucleotide differences per site between two randomly-selected strains.

**Figure 1:**
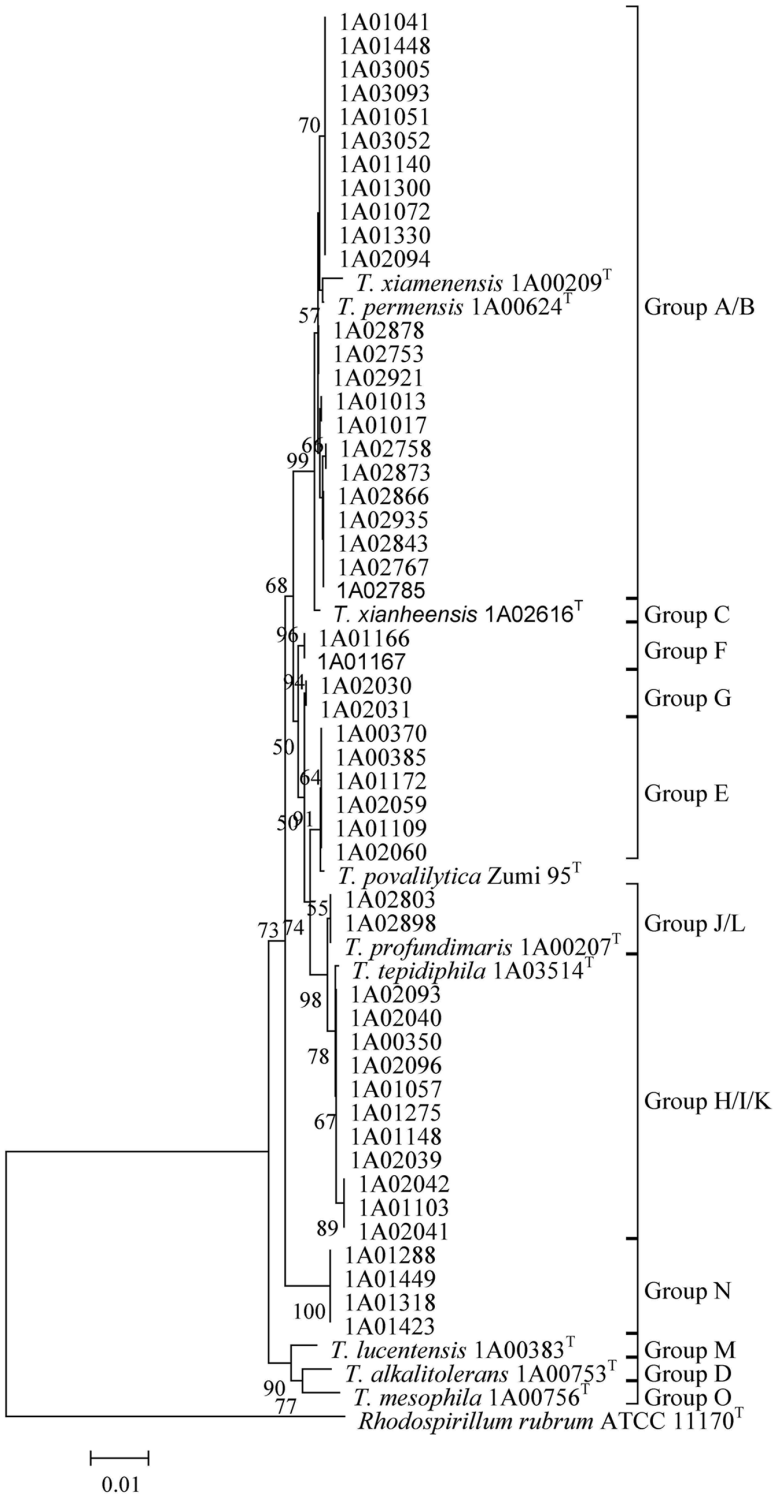
Phylogenetic tree based on the 16S rRNA gene of 59 *Thalassospira* bacteria. The tree was constructed using the neighbor-joining method with MEGA 5.0. Bootstrap values over 50% (1000 replications) were shown at each node. Bar, % estimated substitution. *Rhodospirillum rubrum* ATCC 11170^T^ (NR_074249) was used as the outgroup.

The length of the 16S RNA gene was 1,444 bp, except for three strains named MCCC 1A00383, MCCC 1A00753 and MCCC 1A00753 (1,449 bp). The number of alleles and polymorphic sites of the 16S RNA gene were 20 and 64, respectively; while the proportion of polymorphic sites and nucleotide diversity were 4.42% (4.43%) and 0.012, respectively. The mean G+C content was 55.1 mol%. The range of genetic distance of the 16S RNA gene was 0.000–0.027 (mean 0.008), corresponding to 97.3–100% identity. Measure substitution saturation of the 16S rRNA gene with DAMBE indicated that little saturation existed. The highly conserved 16S rRNA gene was unsuitable for distinguishing 58 *Thalassospira* strains. Despite the low resolution of the 16S rRNA gene, 58 strains were still divided into 11 groups labeled with the group name A to O (Figure 1), the same group names used as in the MLSA phylogenetic tree. In brief, the largest Group A/B contained 25 strains with two type strains, *T. xiamenensis* M-5^T^ and *T. permensis* NBRC106175^T^ (non-valid). The second Group H/I/K consisted of 12 isolates including the type strains *T. tepidiphila* 1-1B^T^. The Group J/L consisted of 3 isolates including the type strain *T. profundimaris* WPO0211^T^. Three type strains, *T. lucentensis* DSM 14000^T^, *T. alkalitolerans* JCM 18968^T^ and *T. mesophila* JCM 18969^T^, located on the root of the phylogenetic tree of the 16S rRNA gene and each represented a group (Group M, D and O). The group C was represented by the type strain *T. xianhensis* P-4^T^. The strains of group E formed a clade with recently described type strain *T. povalilytica* Zumi 95^T^. The rest of three groups (F, G and N) represented potential novel taxa based on the analyses below. However, low bootstrap values at many nodes of the 16S rRNA gene phylogenetic tree implied that topology structure of tree was unstable and the taxonomic affiliation of all strains was inaccurate. Therefore, the 16S rRNA gene was inappropriate for the phylogenetic analysis of the genus *Thalassospira*.

As the 16S rRNA gene was lack of discriminatory power for the closely related strains of the genus *Thalassospira*, six housekeeping genes (*acsA*, *aroE*, *gyrB*, *mutL*, *rpoD* and *trpB*) were utilized for assessing the diversity. The length of six housekeeping genes was from 510 bp for the *rpoD* genes to 918 bp for the *gyrB* gene. The allele numbers of the five housekeeping genes was similar except the *trpB* gene. The *aroE* gene exhibited the highest proportion of polymorphic sites (53.4%), followed by *acsA* (43.2%) and *gyrB* (40.1%). Only 179 polymorphic sites (35.1%) were identified in the *rpoD* gene. It was worthwhile to note that the mean G+C content of six housekeeping gene had significantly different. Similarly, the nucleotide diversity (π, the average number of nucleotide differences per site between two randomly-selected isolates) ranged from 0.119 (*rpoD*) to 0.192 (*aroE*) (Table 2), which showed the different evolution rates of six housekeeping genes in the MLSA scheme. Among the six housekeeping genes, the *aroE* gene exhibited the highest resolution based on the highest mean genetic distance (0.190), whereas *rpoD* displayed the lowest resolution with the lowest mean genetic distance (0.111). Six housekeeping genes showed low *K*a/*K*s ratios of 0.035–0.099, suggesting that they are under negative selection pressure. These results demonstrated that the six housekeeping genes were better than the 16S rRNA gene for differentiating *Thalassospira* bacteria; moreover, the *aroE* gene possessed the highest resolution power among all the tested housekeeping genes.

In this study, 31 unique STs were identified on basis of the six concatenated genes, suggesting high genotypic diversity (Table S3). Of these, 16 STs (27.59%) corresponded to single strain and 14 STs (60.34%) corresponded to two to four isolates. Whereas, the 28th ST (12.07%) was the best represented ST and found in seven different bacteria. Prior to the phylogenetic analysis, the examination of substitution saturation and recombination for six housekeeping genes were subjected using the above-mentioned software, respectively. The measures of the saturation test for single gene with DAMBE version 5.0 shown that the index of *I_SS_* was smaller than the index of *I_SS.C_* in each gene, suggesting no signal of substitution saturation was not found (Table S4). At the same time, the analysis of split decomposition indicated some degree of reticulation (i.e. conflicting phylogenetic signals) for two genes, *acsA* and *rpoD* (Figure S2–S7). The PHI test also demonstrated the presence of recombination for the *acsA* gene (p<0.05) and the *rpoD* gene (p<0.05). Nevertheless, the parallelogram network of the two genes was not very obvious, suggesting that intragenic recombination was not remarkable. Besides, the intergenic recombination was estimated using the LIAN version 3.6. The *I_A_* and standardized index of association (*IS A*) were 5.32 and 0.8634, respectively. Therefore, the null hypothesis of linkage equilibrium (*IS A* = 0) by both parametric and Monte Carlo methods (100 resamplings) was rejected, indicating linkage disequilibrium among the housekeeping genes. As a result, the MLSA analysis was reliable using these housekeeping genes.

In comparison with the phylogenetic tree of the 16S rRNA gene, six phylogenetic trees based on single housekeeping gene sequences had high bootstrap values and presented roughly congruent topology (Figure S8–S13). In detail, the 58 strains were also separated into 15 groups named Group A to O in six phylogenetic trees. Some differences were observed in the topology at the deepest branching points of six phylogenetic trees. For example, the Group D was close to the Group M in the phylogenetic tree of the *mutL* gene; in contrast, it had close relationships to the Group A, B and C in other trees. Interestingly, the strains of Group A and B were mixed in the phylogenetic trees of the *rpoD* gene, this location conflict implies that the acquisition of the *ropD* gene of strain MCCC 1A1300 or MCCC 1A1330 was probably by the lateral gene transfer (LGT) event. Other slight differences were also observed in the six phylogenetic trees, as shown fully in the Figure S8–S13.

### The MLSA analysis

The concatenated genes of six protein-coding genes contained 4,413 bp, 31 alleles and 1,850 polymorphic sites with a mean G+C content of 56.0 mol%. The genetic distance of 58 tested strains ranged from 0.000 to 0.220 with a mean 0.138.

**Figure 2:**
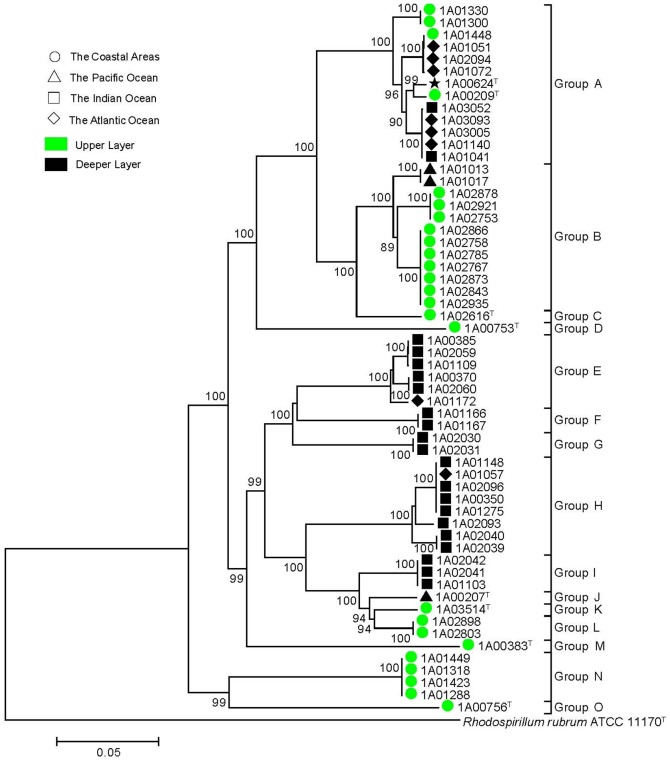
Phylogenetic tree of the MLSA based on six concatenated housekeeping genes of the genus *Thalassospira*. The tree was constructed using the neighbor-joining method with MEGA 5.0. Bootstrap values over 50% (1000 replications) were shown at each node. Bar, % estimated substitution. *Rhodospirillum rubrum* ATCC 11170^T^ (NC_007643) was used as the outgroup. Meantime, the isolated areas and water depth of the *Thalassospira* bacteria were divided artificially and marked by different symbols or colors.

The concatenated genes tree showed a similar topology to the individual gene tree including 15 Groups from A to O with very high bootstrap support (Figure 2). Specifically, the Group A was the largest and had five branches including 13 strains with two type strains *T. xiamenensis* M-5^T^ and *T. permensis* NBRC106175^T^ (non-valid). The second largest Group B was separated into three different branches with 12 strains. The Group E contained six strains with three branches including five genetic types. The Group H was split into three branches corresponding to three genetic types with eight strains. The six type strains, *T. xianhensis* P-4^T^, *T. alkalitolerans* JCM 18968^T^, *T. profundimaris* WP0211^T^, *T. tepidiphila* 1-1B^T^, *T. lucentensis* DSM 14000^T^, and *T. mesophila* JCM 18969^T^, formed respectively a separate group marked as Group C, D, J, K, M and O. The rest of five minority groups (Group F, G, I, L and N) and Group B, E and H, cannot be allocated to any previously described species. Therefore, they are new taxon, representing eight potential novel species.

### Correlation analysis between similarities of the MLSA and DDH, ANI, and the evaluation of radiation times

**Figure 3:**
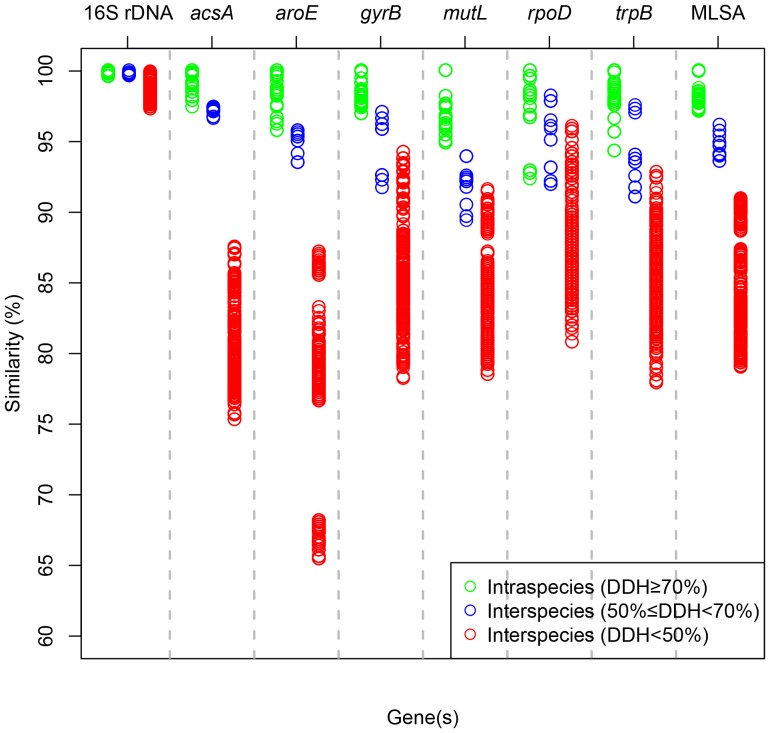
Intraspecies and interspecies identity of 16S rRNA gene and housekeeping gene(s) of the genus *Thalassospira* bacteria. The identity values of each other were displayed with three color circles (intraspecies: green, interspecies: blue/red).

In order to further confirm the accuracy of the MLSA analysis, the DDH values of the 16 representative strains of the 13 groups except the two newly described species (Group D and Group O) were calculated in silico from draft genomes (Table S5). Considering the 70% DDH values as a gold standard for the species boundary in prokaryote taxonomy, 16 strains of the *Thalassospira* genus were classified into the 13 species corresponding to the 13 group of the MLSA analysis. And then, the intraspecies and interspecies similarities of the individual gene and the concatenated genes among 58 strains were determined based on the species given by DDH values and shown in Figure 3. Disappointingly, identity values of the intraspecies and the interspecies partially overlapped in the most individual genes except the *mutL* gene. The overlap of identity values was encountered by the strains between Group B and C, and among the strains of Group J, K and L, which shared the high interspecies identity values, with the low intraspecies identity values existing in the strains within Group A, B or H. In contrast, identity values of the concatenated genes exhibited a distinct demarcation (96.16–97.12%) between intraspecies and interspecies. Consequently, compared with the single gene, the MLSA has a great advantage in distinguishing the *Thalassospira* bacteria.

The correlation between the identity of the concatenated genes and DDH in 16 strains was determined by a nonlinear simulate analysis method. As can be seen in Figure S14, the identity values of the six concatenated housekeeping genes was highly correlated (R^2^ = 0.9661) with the DDH values. Based on the simulative logarithmic equation (y = 11.87*lnx+46.58), DDH value of 70% was equivalent to 97% identity of the MLSA, suggesting that 97% identity of the MLSA as interspecies gap can be used for the cut-off of the species for the *Thalassospira* bacteria. In addition, as shown in Table S6, the ANIm values of interspecies ranged from 83.34% to 95.76%, whereas the values of intraspecies were from 97.20% to 97.93%. These ANIm values were in accordance with standard ANI criteria for species identity (95–96%) [29].

The radiation times of each other in 58 strains of the genus *Thalassospira* were estimated using the rate of synonymous substitution for six housekeeping genes. On interspecies level, the radiation time between the strain MCCC 1A02803 (representing the Group L) and the strain MCCC 1A03514 (representing the Group K) was the lowest, about 22.21 million years. By contrast, these two strains had highly related genome, with 64.3% DDH values. This result indicates that the differentiation of two young species started at 22.21 million years ago. On intraspecies level, the highest radiation time between the strain MCCC 1A00624 and MCCC 1A01300 from the same group was 19.30 million years, the DDH value of the two strains was 80.7%. Certainly, these two strains would be divided into different species only several millions years ago. A common ancestor of the strains in the genus *Thalassospira* might occur 161.48 million years ago (Figure S15).

### Biogeography analysis

In this study, the geographical locations of the 57 strains of the genus *Thalassospira* except of *T. permensis* NBRC106175^T^ covered a wide range of marine environments. According to the origin of these strains, four large areas, the coastal area (circle), the Pacific Ocean (triangle), the Indian Ocean (square) and the Atlantic Ocean (diamond), were marked using four symbols in MLSA phylogenetic tree, respectively. As shown in Figure 2, strains from the coastal area and the Indian Ocean clustered respectively together to some extent; for instance strains of Group B, L and N were from coastal area, Group E, F, G, H and I were from Indian Ocean; whereas the strains from the Pacific Ocean and the Atlantic Ocean scattered in several groups in the MLSA phylogenetic tree.

In addition, to explore the affection of seawater depth, the habitats were artificially partitioned into the upper layer (0–100 m water depth) and the deeper layer (668–4,480 m water depth) and marked in green and black, respectively, in the MLSA phylogenetic tree (Figure 2). Intriguingly, the most strains tended to cluster together to a certain degree according to the habitats depth. In detail, the strains of the Group B (not including MCCC 1A01013 and MCCC 1A01017) and the Group C were all isolated from the upper layer (marked in Green) and gathered together, while the strains of the Group E, F, G, H, I and J came from the deeper layer (marked in Black) got together. These results indicate that the water depth is one of the key environmental factors for affecting the differentiation of the *Thalassospira* bacteria.

## Discussion

Bacteria of the genus *Thalassospira* frequently occur in various marine environments,such as seawater, sediment, halobios from every oceans and seas, and frequently retrieved from the bacterial communities enriched using PAHs or crude oil. Recently, they were found to involve the phosphorus cycling in marine environments [13], [14]. The accumulations of isolates of this genus urge the systematic phylogeny analyses. In this report, the genetic diversity and geographic distribution of the *Thalassospira* bacteria were analyzed for the first time using many approaches including the MLSA with six protein-coding genes, DDH and ANI.

In this study, we demonstrated that MLSA was a powerful method for the discrimination, classification and phylogenetic analysis of the *Thalassospira* bacteria, which cannot be distinguished by the 16S rRNA gene. The similar situations were frequently encountered in other genera, such as *Bacillus*, *Vibrio*, *Pseudomonas*, *Enterobacter* and *Aeromonas* etc. [20], [24], [25], [42], [43]. Recently, the single housekeeping gene (*gyrB*, *rpoB*, *pyrE*, *aroE* and so on) replaced the 16S rRNA gene and was widely used as a phylogentic marker for differentiating the closely related strains [21], [24], [43], [44]. Due to the horizontal gene transfer [45] and genetic recombination [25], single housekeeping gene may distort the phylogenetic tree. In this report, for example, the strain MCCC 1A02616 occurred in the horizontal gene transfer in the *rpoD* gene; while the MLSA based on several housekeeping genes are more reliable. In this study, the 58 strains of the genus *Thalassospira* were split into 15 groups using the MLSA; each of the seven groups (Group A, C, D, J, K, M and O) represented by a described type strain, and eight groups (Group B, E, F, G, H, I, L and N) represented a potential novel species awaiting for further classification of polyphasic taxonomy (Six strains of the Group E probably belong to recently described species *T. povalilytica*). In addition, DDH value of 70% was used as the gold standard for bacterial species delineation [46]. In this report, we chose 16 representative strains from different groups for DDH and ANI calculation. As a result, the DDH values verified the results of MLSA analysis, and in addition defined the interspecies gap for the genus *Thalassospira*, with an identity range from 96.16% to 97.12% based on six housekeeping genes. The interspecies gap of ANI value ranged from 95.76% to 97.20%. In the recent studies, the classification and the genetic diversity of the closely related strains in the intragenus were explored by the MLSA combined with the DDH [21], [42], [47], [48]. Therefore, the MLSA is an effective, straightforward, reproducible and comparable tool to explore the taxonomy of the bacteria, to study the diversity of the strains from various environments, to understand evolution of species.

The isolated origins of all culturable strains in our lab laboratory and the NCBI database indicate that they should be the obligate marine bacteria though the non-validly published species, *T. permensis* SMB34^T^, was isolated from primitive technogeneous soil formed on salt-mine spoils at the Verchnekamsk deposit of potassium magnesium salts (Berezniki, Perm region, Russia). The geographic distribution patterns showed that the bacteria from the coastal area clustered together to some extent, as well as those from the Indian Ocean. However, the strains from the Pacific Ocean and the Atlantic Ocean scattered in the MLSA phylogenetic tree (Fig. 3). Many similar studies have been reported in recent years. The bacteria of *Bacillus pumilus* group from marine and terrestrial environments clustered respectively based on the *gyrB* gene phylogenetic tree [24]. Likewise, Khan *et al*. discovered that the oceanic *Pesudomonas aeruginosa* strains trended distinct to cluster from those of other *P. aeruginosa* strains [44]. In addition, The *Vibrio parahaemolyticus* bacteria from different epidemiological sources in Thailand revealed a degree of clustering in phylogenetic analysis [49]. The inherent mechanism of the intriguing clustering in different origin isolates remains unclear, and then explores further in future investigations.

Obviously in the tree (Fig. 3), two ecotypes were separated from each other according to the water depths. Thus, the surface ecotype and deep-sea ecotype naturally formed in the vast marine system. Our results were consistent with the previous reports. For example, the marine bacterium *Alteromonas macleodii* are found to be generally clustered with the depth in the water column from which the isolate originated [50]. Klochko *et al* found that peculiarities of *A. macleodii* strains reflected their deep/surface habitation rather than geographical distribution [51]. Similarly, Tarasov *et al* exposed significant differences between deep and shallow-water hydrothermal vent communities [52].

In summary, we reported the diversity and biogeography of *Thalassospira* bacteria from diverse marine environments for the first time, based on the MLSA, DDH and ANI. Fifteen distinct lineages corresponding to seven known and eight potential novel taxonomic groups were revealed, and the interspecies gap of MLSA for the genus *Thalassospira* is 96.16–97.12%. Interestingly, the *Thalassospira* bacteria exhibited surface and deep ecotypes according to the water depths. These results help to understand their adaptive evolution in marine environments. Further polyphasic characterization and comparative genomic analysis are just under study to understand their role in marine environments.

## Supporting Information

Figure S1 **The map of geographical distribution of the 58 strains from various marine environments.** Each red dot represents a strain, some dots overlapped.
(DOCX)

Figure S2 **Split decomposition analysis of the** ***acsA*** **gene.**
(DOCX)

Figure S3 **Split decomposition analysis of the** ***aroE*** **gene.**
(DOCX)

Figure S4 **Split decomposition analysis of the** ***gyrB*** **gene.**
(DOCX)

Figure S5 **Split decomposition analysis of the** ***mutL*** **gene.**
(DOCX)

Figure S6 **Split decomposition analysis of the** ***rpoD*** **gene.**
(DOCX)

Figure S7 **Split decomposition analysis of the** ***trpB*** **gene.**
(DOCX)

Figure S8 **Phylogenetic tree based on the** ***acsA*** **gene.** The tree was constructed using the neighbor-joining method with MEGA 5.0. Bootstrap values over 50% (1000 replications) were shown at each node. Bar, % estimated substitution. The bacteria of *Rhodospirillum rubrum* ATCC 11170^T^ (NC_007643) was used as the outgroup.
(DOCX)

Figure S9 **Phylogenetic tree based on the** ***aroE*** **gene.** The tree was constructed using the neighbor-joining method with MEGA 5.0. Bootstrap values over 50% (1000 replications) were shown at each node. Bar, % estimated substitution. The bacteria of *Rhodospirillum rubrum* ATCC 11170^T^ (NC_007643) was used as the outgroup.
(DOCX)

Figure S10 **Phylogenetic tree based on the** ***gyrB*** **gene.** The tree was constructed using the neighbor-joining method with MEGA 5.0. Bootstrap values over 50% (1000 replications) were shown at each node. Bootstrap values over 50% (1000 replications) were shown at each node. Bar, % estimated substitution. The bacteria of *Rhodospirillum rubrum* ATCC 11170^T^ (NC_007643) was used as the outgroup.
(DOCX)

Figure S11 **Phylogenetic tree based on the** ***mutL*** **gene.** The tree was constructed using the neighbor-joining method with MEGA 5.0. Bootstrap values over 50% (1000 replications) were shown at each node. Bootstrap values over 50% (1000 replications) were shown at each node. Bar, % estimated substitution. The bacteria of *Rhodospirillum rubrum* ATCC 11170^T^ (NC_007643) was used as the outgroup.
(DOCX)

Figure S12 **Phylogenetic tree based on the** ***rpoD*** **gene.** The tree was constructed using the neighbor-joining method with MEGA 5.0. Bootstrap values over 50% (1000 replications) were shown at each node. Bar, % estimated substitution. The bacteria of *Rhodospirillum rubrum* ATCC 11170^T^ (NC_007643) was used as the outgroup.
(DOCX)

Figure S13 **Phylogenetic tree based on the** ***trpB*** **gene.** The tree was constructed using the neighbor-joining method with MEGA 5.0. Bootstrap values over 50% (1000 replications) were shown at each node. Bar, % estimated substitution. The bacteria of *Rhodospirillum rubrum* ATCC 11170^T^ (NC_007643) was used as the outgroup.
(DOCX)

Figure S14 **The correlation of the DDH and MLSA identity of the 16** ***Thalassospira*** **bacteria.**
(DOCX)

Figure S15 **The clustering tree of the DDH values and Radiation time of strains in the three clusters.**
(DOCX)

Table S1 Characteristics of the primers used in this study.
(DOCX)

Table S2 GenBank accession numbers of all strains used in this study.
(DOCX)

Table S3 Allelic profiles of all strains used in this study.
(DOCX)

Table S4 Nucleotide diversity, mearsure substitution saturation and the PHI test for single gene.
(DOCX)

Table S5 The identity matrix of DDH and MLSA in the 16 *Thalassospira* bacteria.
(DOCX)

Table S6 The identity matrix of ANIm in the 16 *Thalassospira* bacteria.
(DOCX)
