# Supplementary figures and images for: Multilocus Sequence Analysis for Assessment of Phylogenetic Diversity and Biogeography in Thalassospira Bacteria from Diverse Marine Environments
Source: PLoS One. 2014 Sep 8;9(9):e106353. doi: 10.1371/journal.pone.0106353 (PMC4157779; doi:10.1371/journal.pone.0106353)

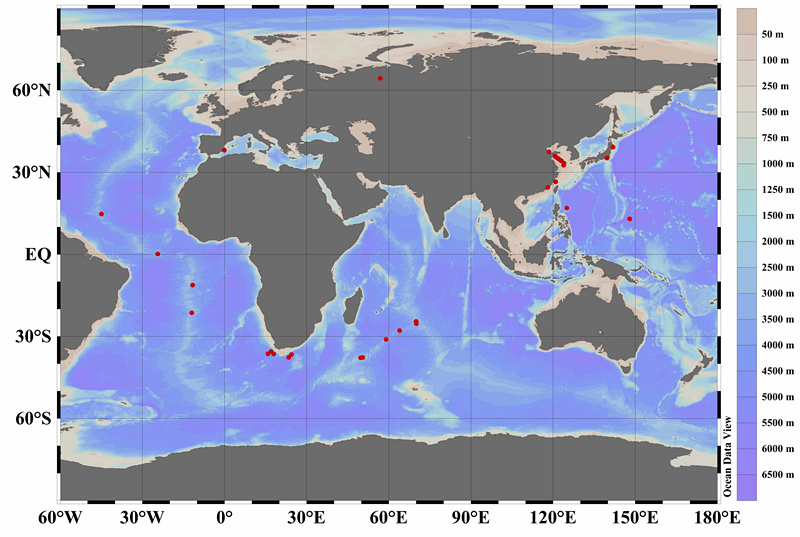


Figure S1. The map of geographical distribution the 58 strains from various marine environments.

Supplement: Figure S1 — The map of geographical distribution of the 58 strains from various marine environments. Each red dot represents a strain, some dots overlapped. (DOCX) [file pone.0106353.s001.docx]

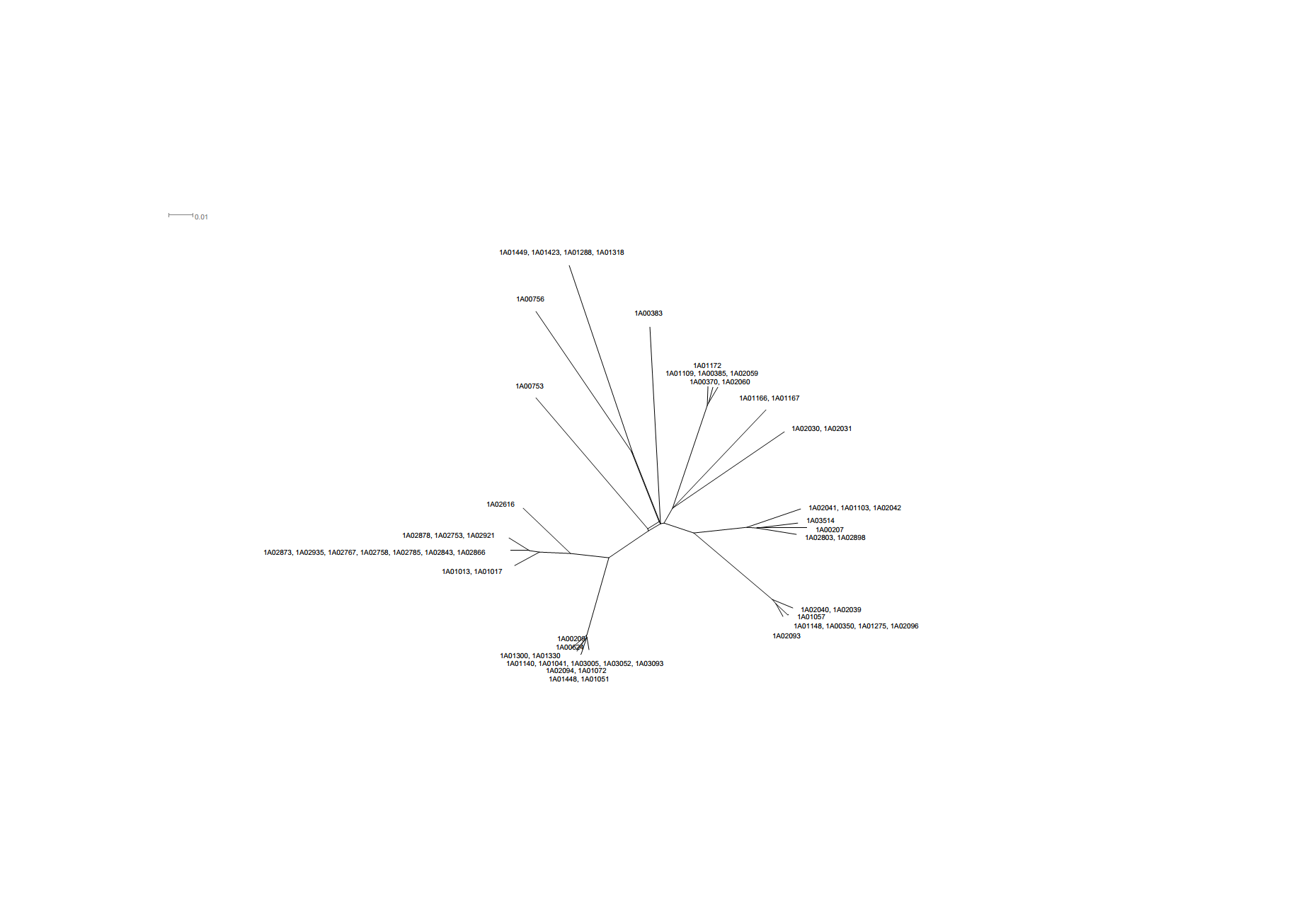
Figure S3. Split decomposition analysis of the *aroE* gene.

Supplement: Figure S3 — Split decomposition analysis of the aroE gene. (DOCX) [file pone.0106353.s003.docx]

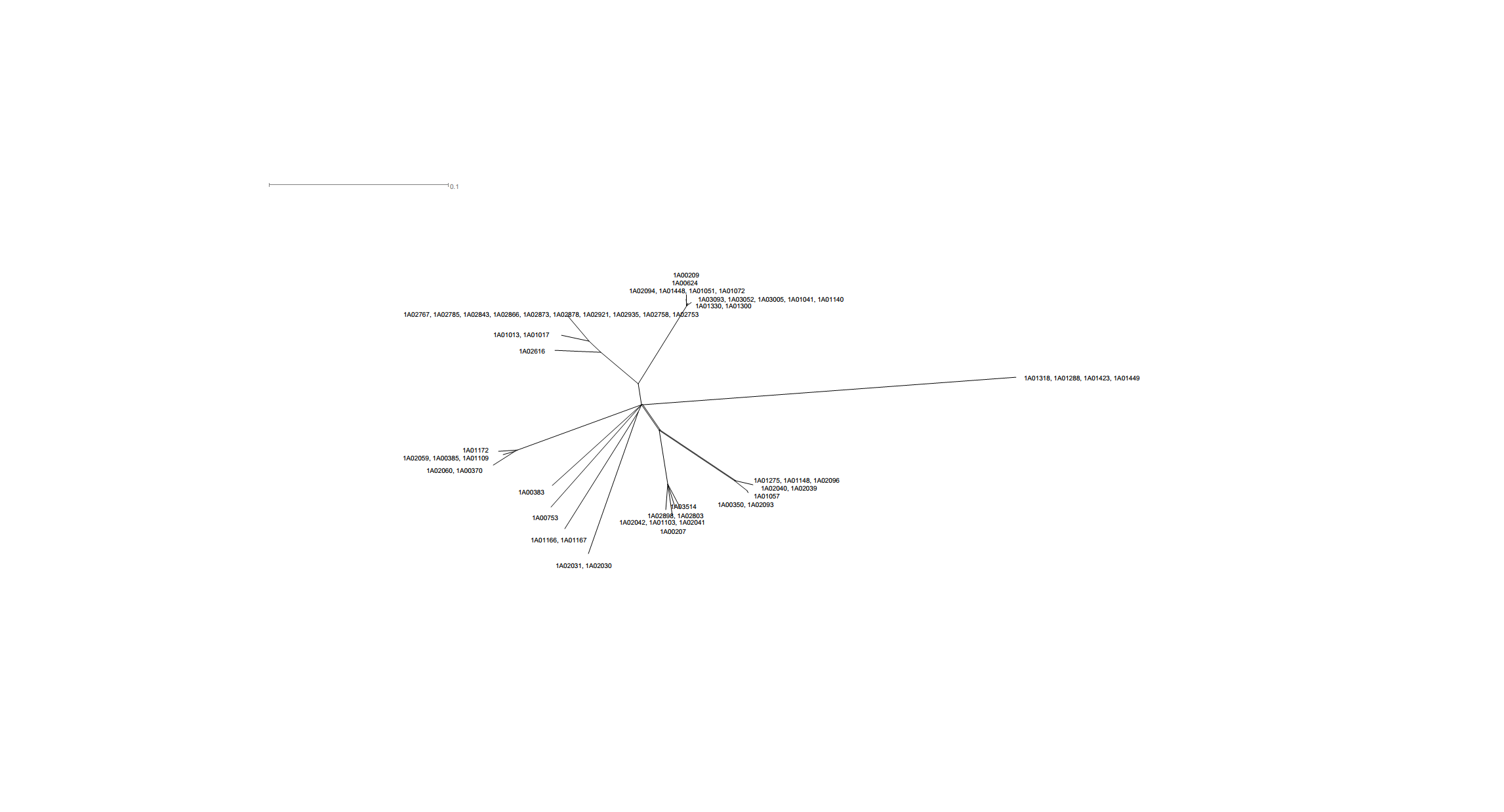
Figure S4. Split decomposition analysis of the *gyrB* gene.

Supplement: Figure S4 — Split decomposition analysis of the gyrB gene. (DOCX) [file pone.0106353.s004.docx]

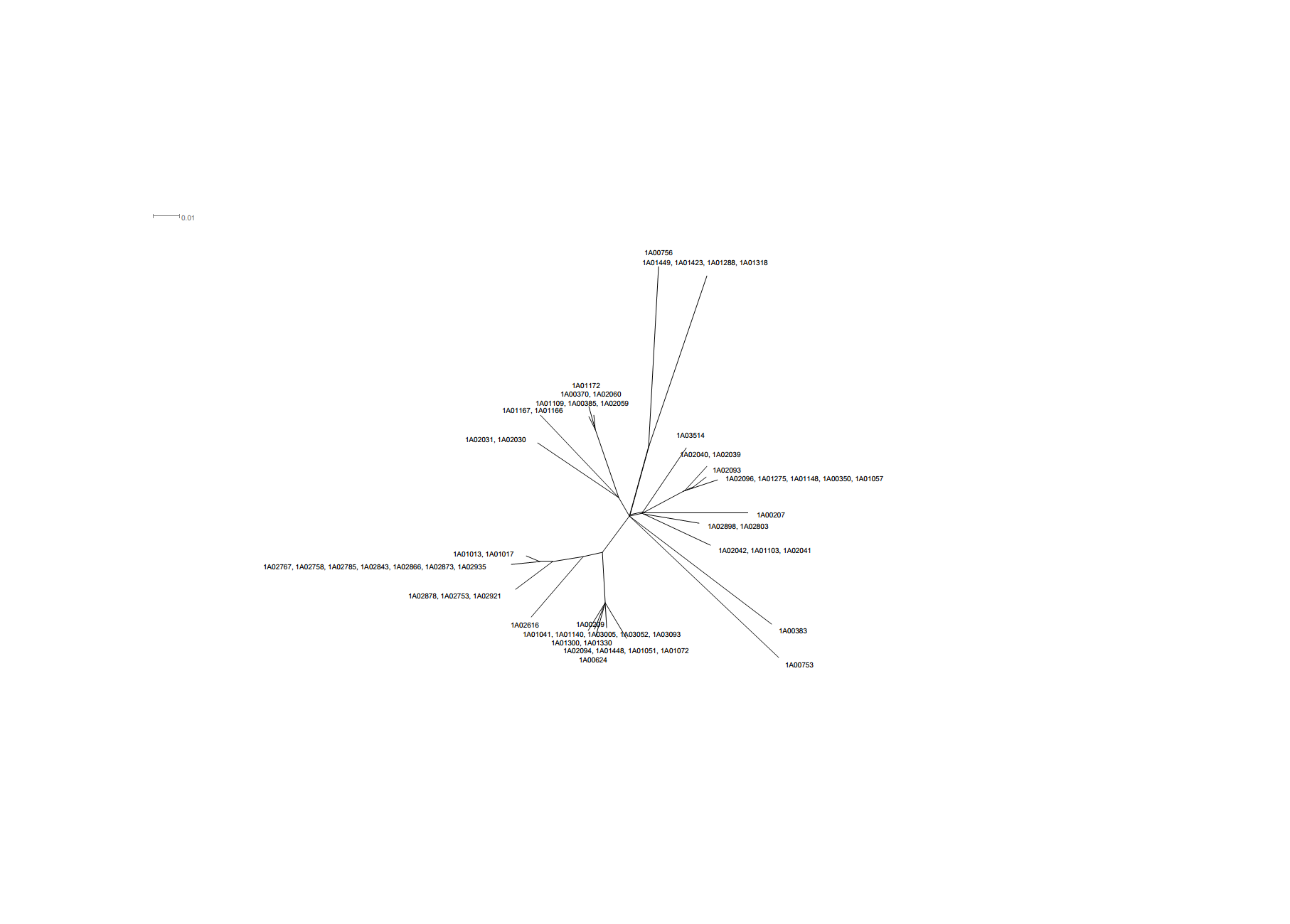
Figure S5. Split decomposition analysis of the *mutL* gene.

Supplement: Figure S5 — Split decomposition analysis of the mutL gene. (DOCX) [file pone.0106353.s005.docx]

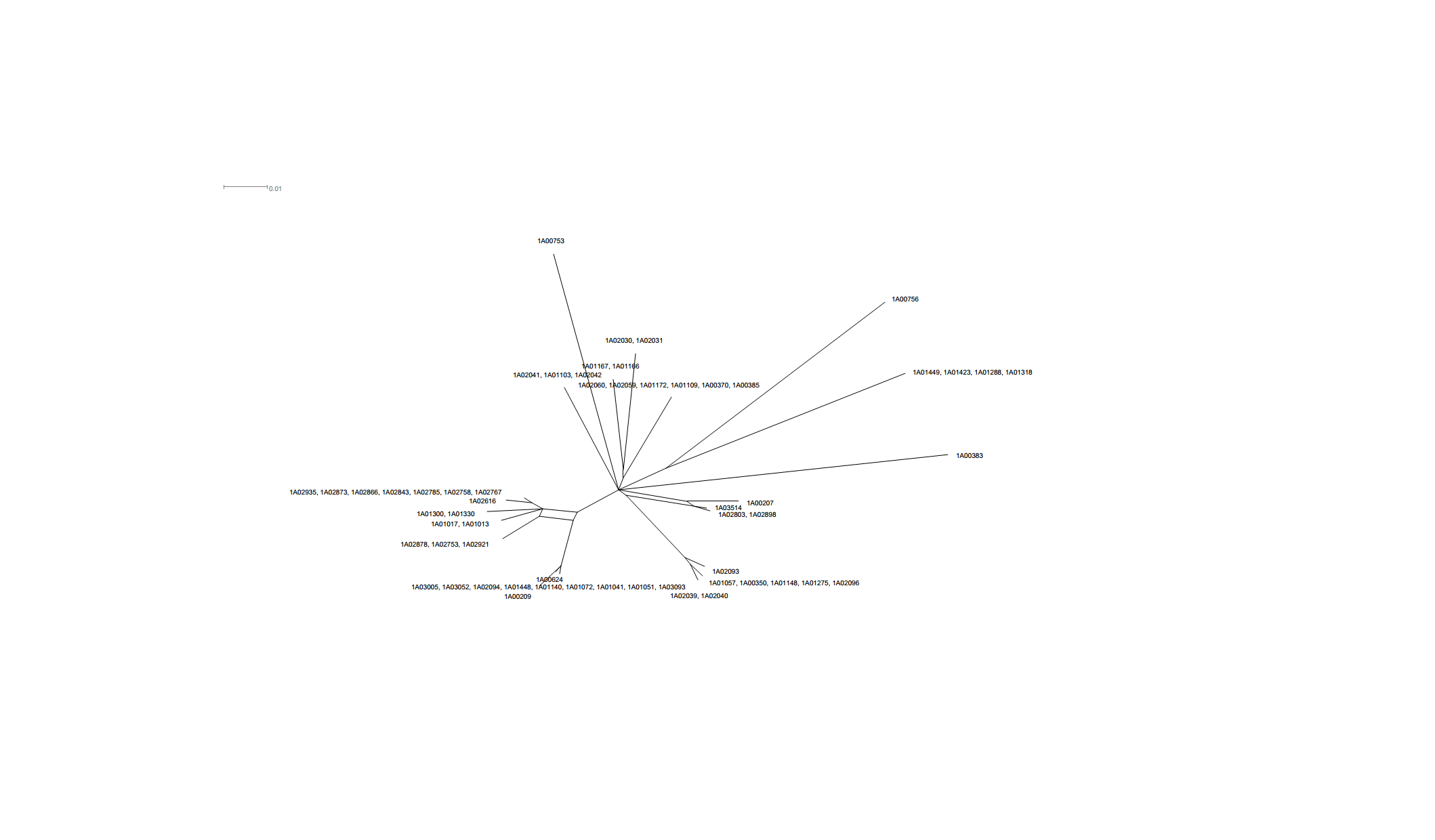
Figure S6. Split decomposition analysis of the *rpoD* gene.

Supplement: Figure S6 — Split decomposition analysis of the rpoD gene. (DOCX) [file pone.0106353.s006.docx]

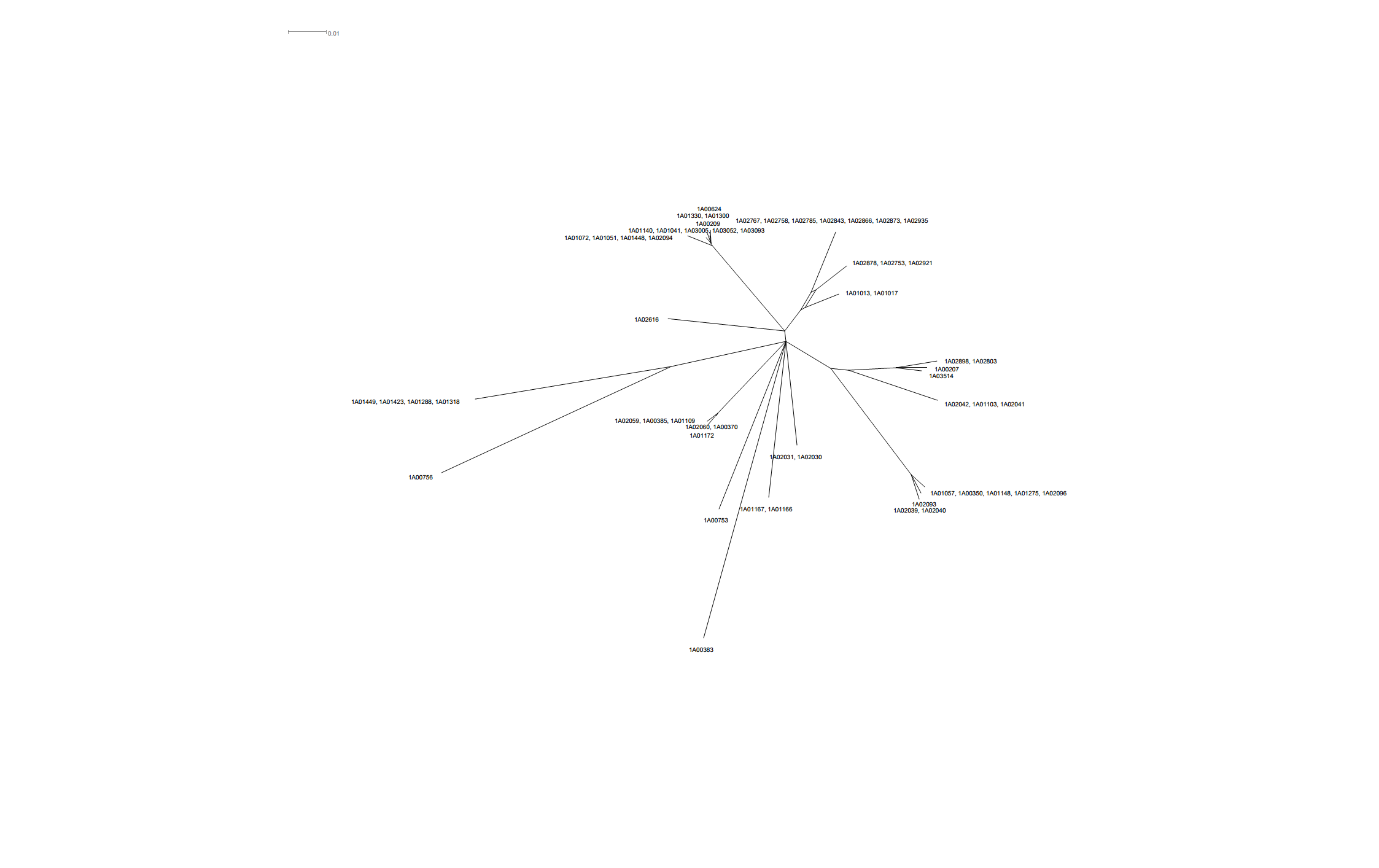
Figure S7. Split decomposition analysis of the *trpB* gene.

Supplement: Figure S7 — Split decomposition analysis of the trpB gene. (DOCX) [file pone.0106353.s007.docx]

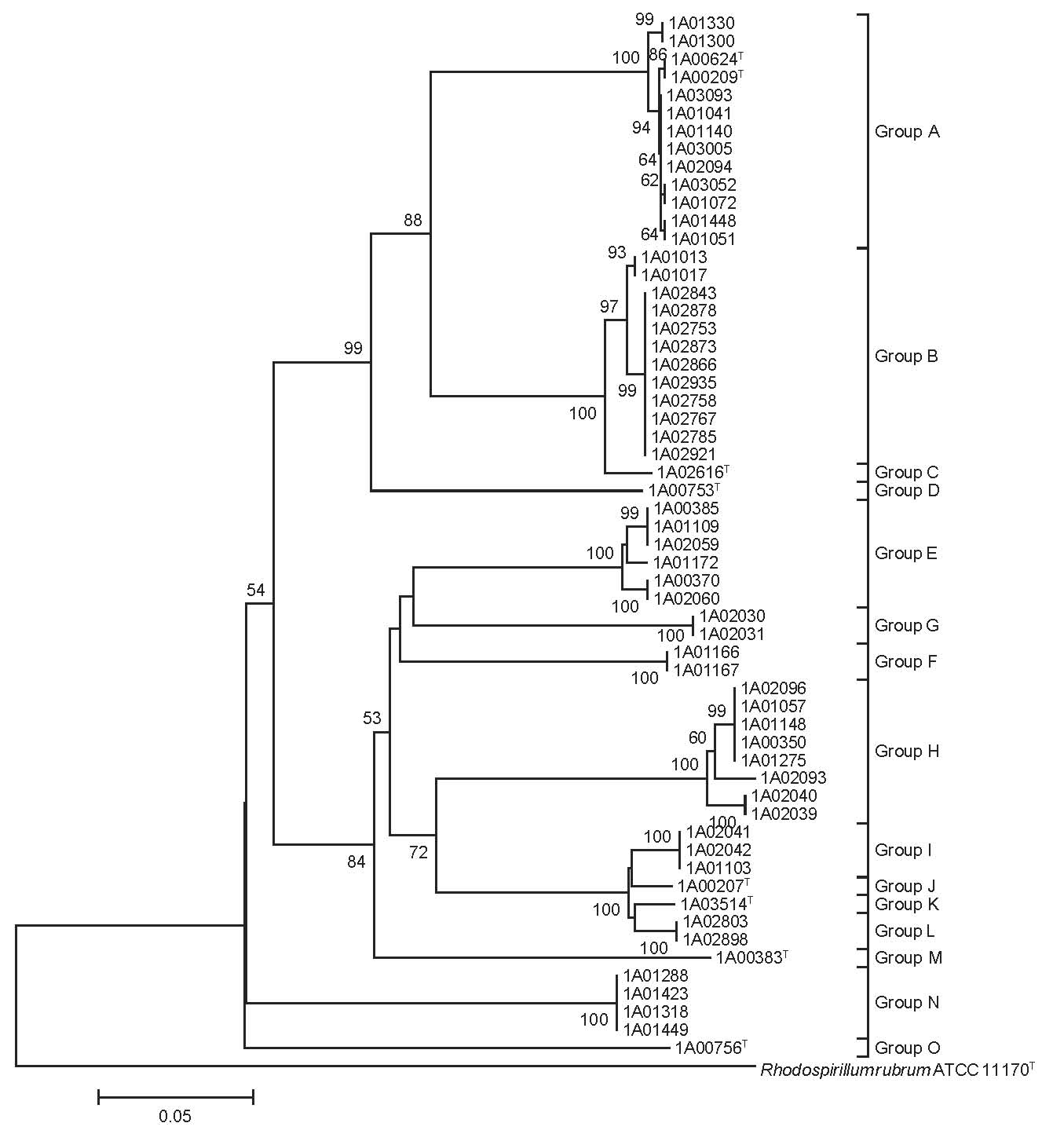


Figure S8. Phylogenetic tree based on the *acsA* gene

Supplement: Figure S8 — Phylogenetic tree based on the acsA gene. The tree was constructed using the neighbor-joining method with MEGA 5.0. Bootstrap values over 50% (1000 replications) were shown at each node. Bar, % estimated substitution. The bacteria of Rhodospirillum rubrum ATCC 11170T (NC_007643) was used as the outgroup. (DOCX) [file pone.0106353.s008.docx]

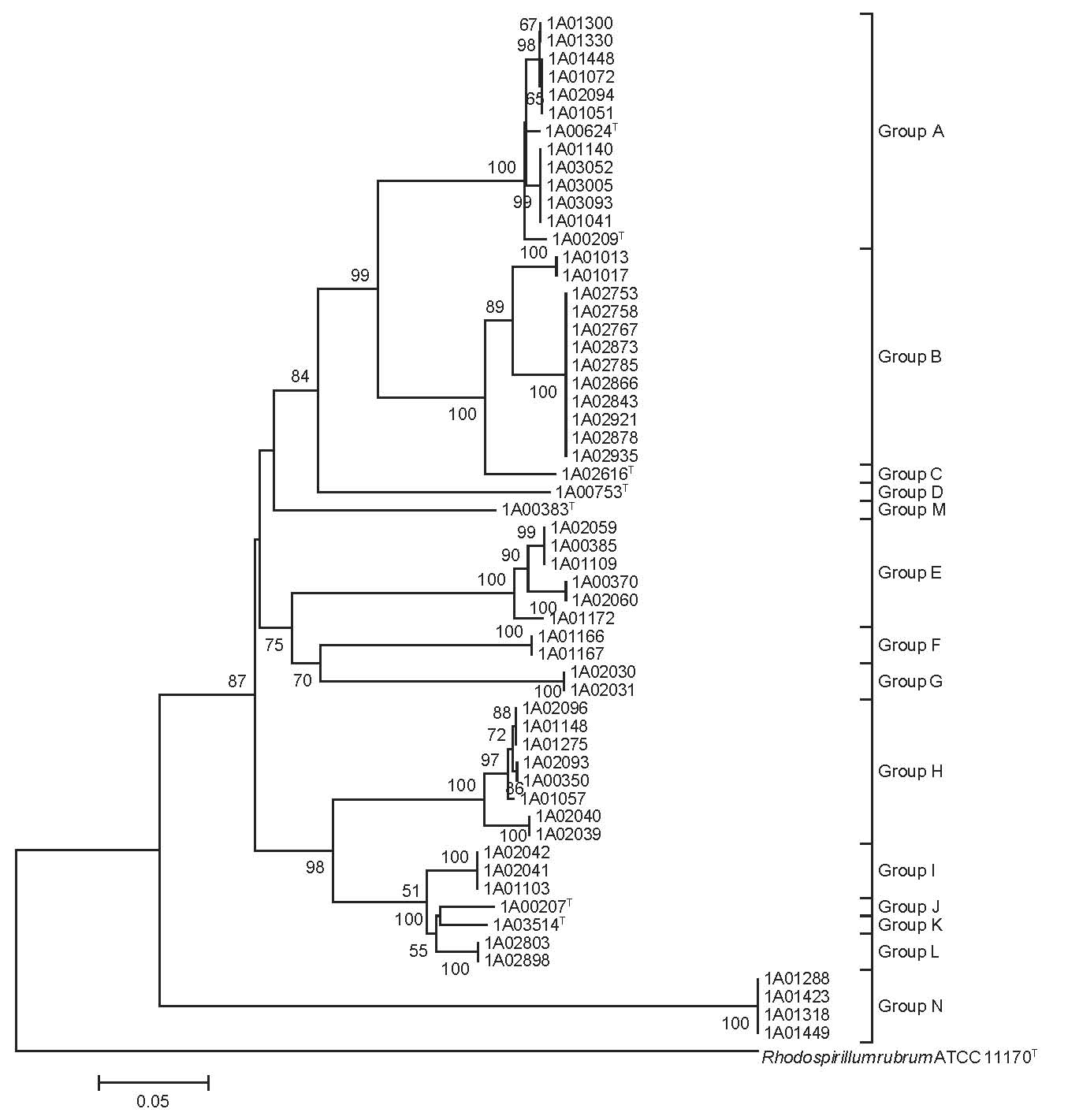


Figure S9 Phylogenetic tree based onthe *aroE* gene.

Supplement: Figure S9 — Phylogenetic tree based on the aroE gene. The tree was constructed using the neighbor-joining method with MEGA 5.0. Bootstrap values over 50% (1000 replications) were shown at each node. Bar, % estimated substitution. The bacteria of Rhodospirillum rubrum ATCC 11170T (NC_007643) was used as the outgroup. (DOCX) [file pone.0106353.s009.docx]

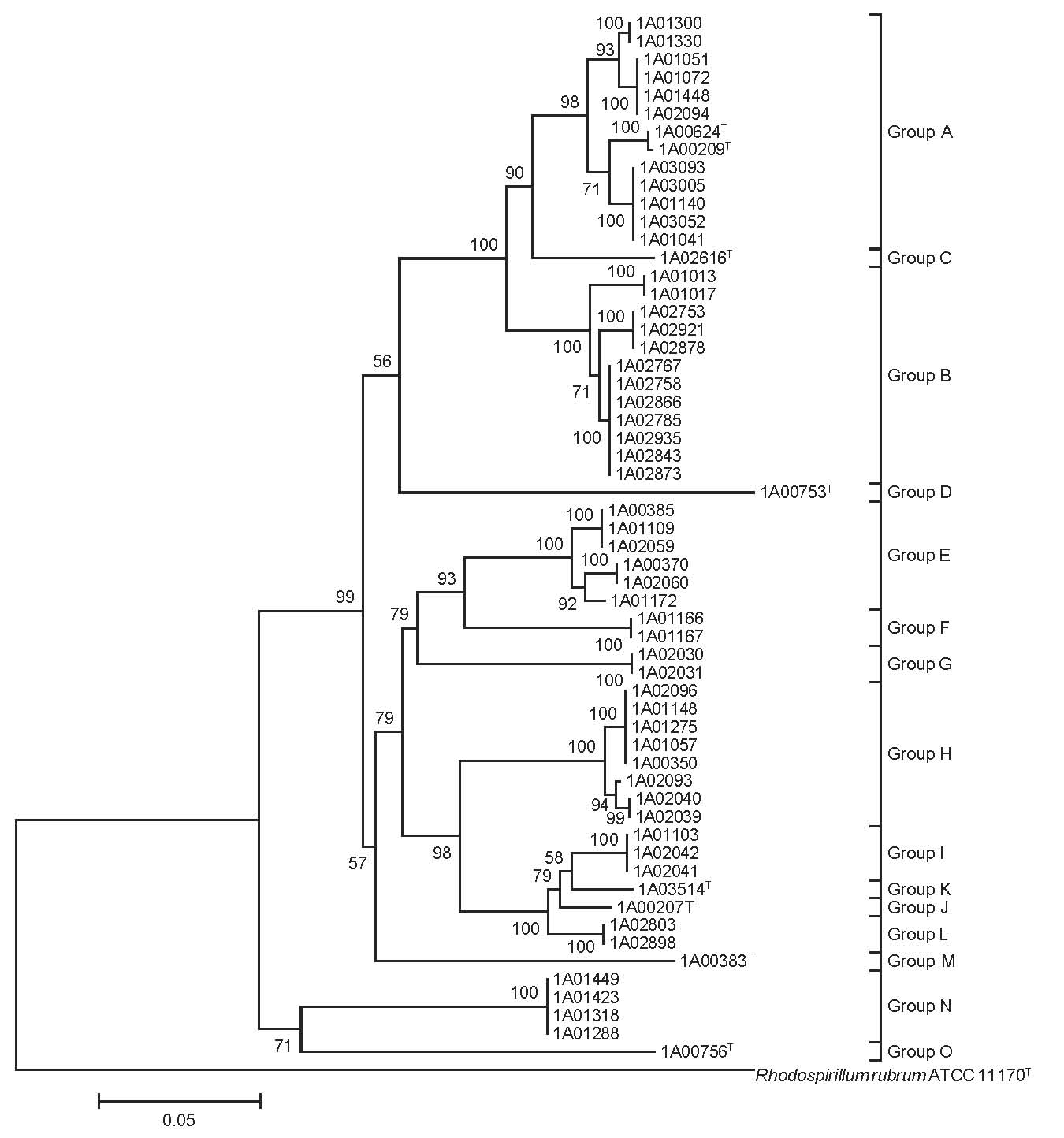


Figure S10. Phylogenetic tree based onthe *gyrB* gene.

Supplement: Figure S10 — Phylogenetic tree based on the gyrB gene. The tree was constructed using the neighbor-joining method with MEGA 5.0. Bootstrap values over 50% (1000 replications) were shown at each node. Bootstrap values over 50% (1000 replications) were shown at each node. Bar, % estimated substitution. The bacteria of Rhodospirillum rubrum ATCC 11170T (NC_007643) was used as the outgroup. (DOCX) [file pone.0106353.s010.docx]

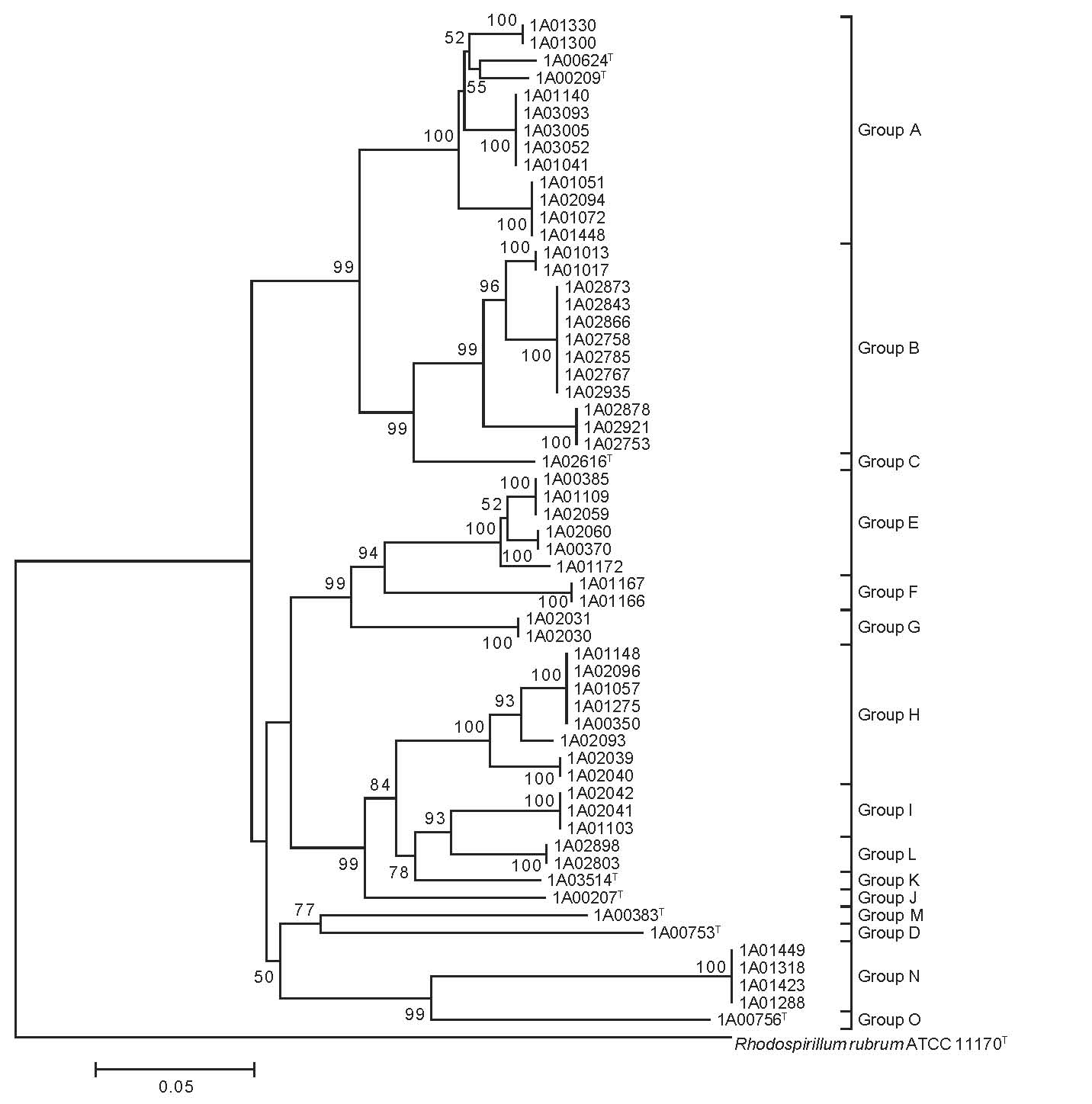


Figure S11. Phylogenetic tree based on the *mutL* gene.

Supplement: Figure S11 — Phylogenetic tree based on the mutL gene. The tree was constructed using the neighbor-joining method with MEGA 5.0. Bootstrap values over 50% (1000 replications) were shown at each node. Bootstrap values over 50% (1000 replications) were shown at each node. Bar, % estimated substitution. The bacteria of Rhodospirillum rubrum ATCC 11170T (NC_007643) was used as the outgroup. (DOCX) [file pone.0106353.s011.docx]

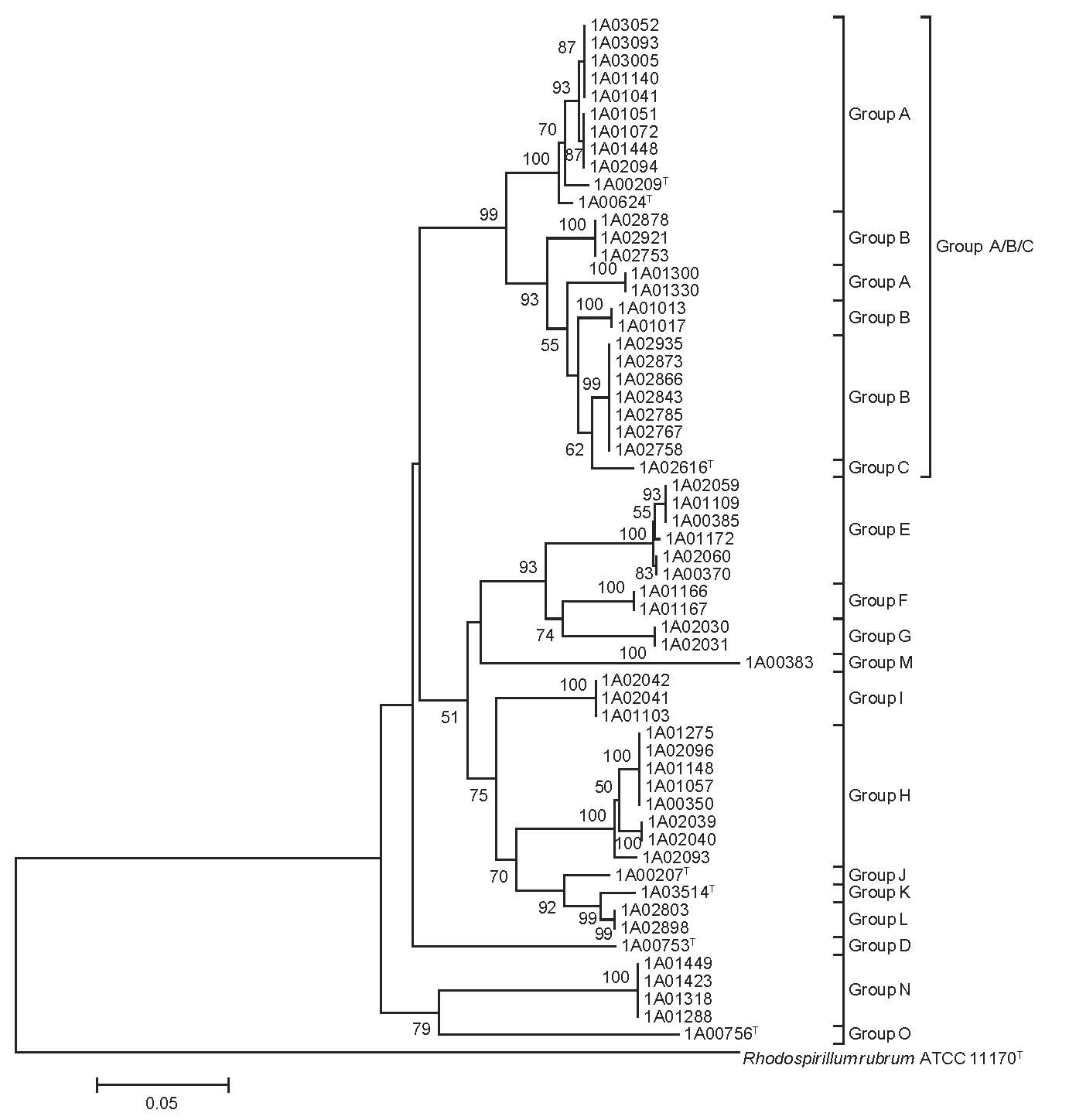


Figure S12. Phylogenetic tree based on the *rpoD* gene.

Supplement: Figure S12 — Phylogenetic tree based on the rpoD gene. The tree was constructed using the neighbor-joining method with MEGA 5.0. Bootstrap values over 50% (1000 replications) were shown at each node. Bar, % estimated substitution. The bacteria of Rhodospirillum rubrum ATCC 11170T (NC_007643) was used as the outgroup. (DOCX) [file pone.0106353.s012.docx]

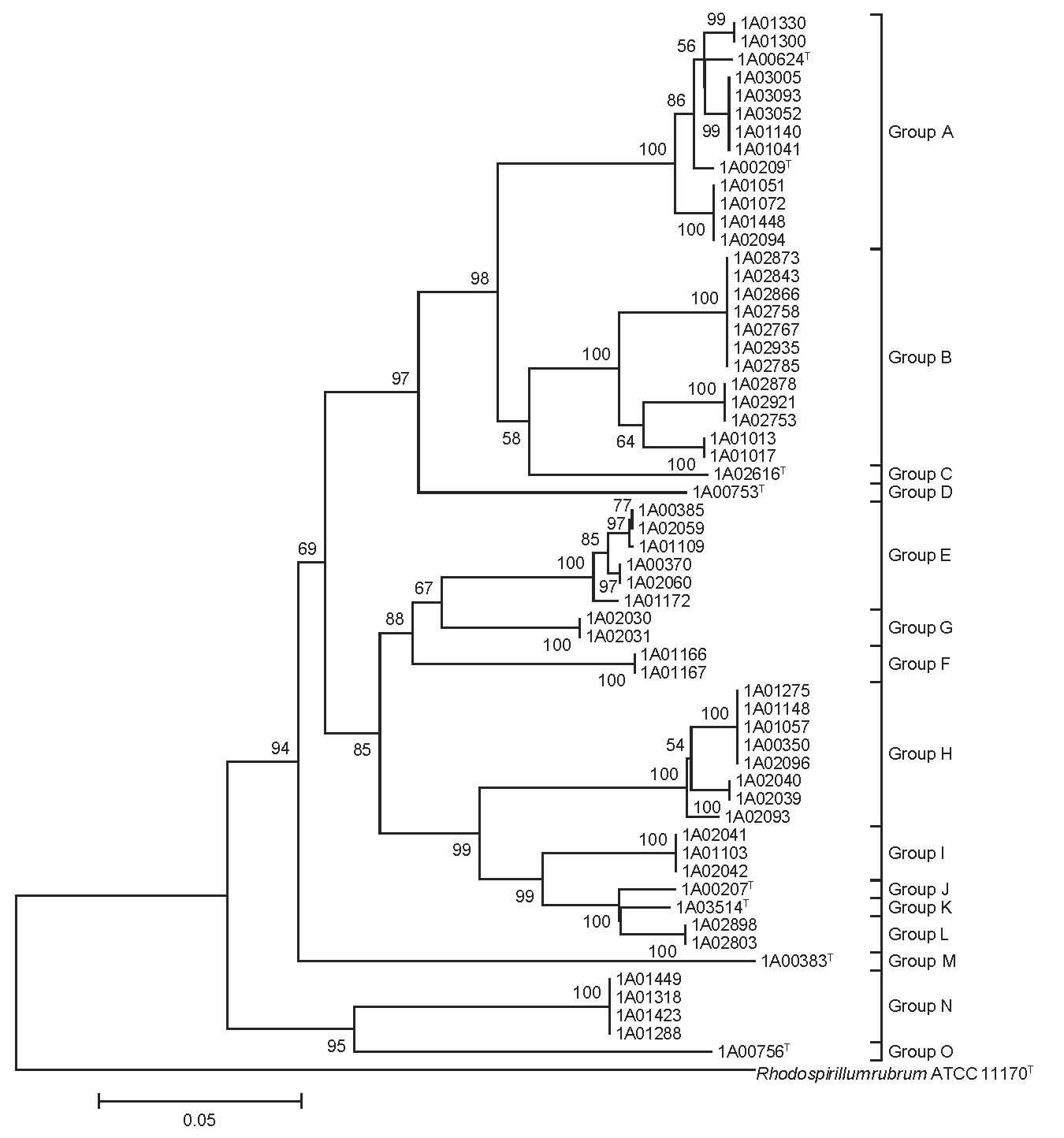


Figure S13. Phylogenetic tree based on the *trpB* gene.

Supplement: Figure S13 — Phylogenetic tree based on the trpB gene. The tree was constructed using the neighbor-joining method with MEGA 5.0. Bootstrap values over 50% (1000 replications) were shown at each node. Bar, % estimated substitution. The bacteria of Rhodospirillum rubrum ATCC 11170T (NC_007643) was used as the outgroup. (DOCX) [file pone.0106353.s013.docx]

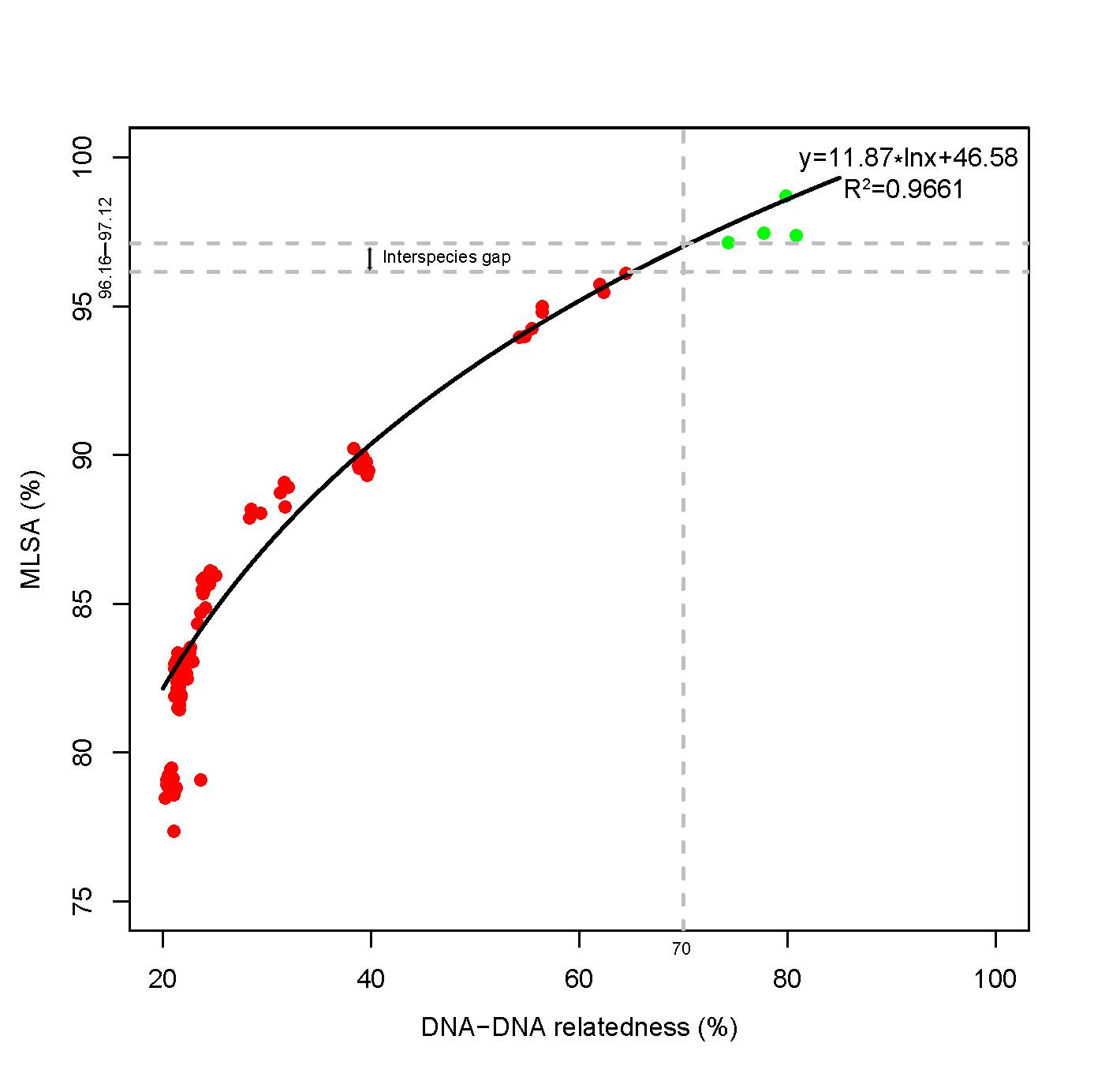


Figure S14. The correlation of the DDH and MLSA identity of the 16 *Thalassospira* bacteria.

Supplement: Figure S14 — The correlation of the DDH and MLSA identity of the 16 Thalassospira bacteria. (DOCX) [file pone.0106353.s014.docx]
