# Supplementary material for: Multilocus Sequence Analysis for Assessment of Phylogenetic Diversity and Biogeography in Thalassospira Bacteria from Diverse Marine Environments
Source: PLoS One. 2014 Sep 8;9(9):e106353. doi: 10.1371/journal.pone.0106353 (PMC4157779; doi:10.1371/journal.pone.0106353)
Supplement: Figure S2 — Split decomposition analysis of the acsA gene. (DOCX) [file pone.0106353.s002.docx]

Figure S2. Split decomposition analysis of the *acsA* gene.
