# Supplementary material for: Multilocus Sequence Analysis for Assessment of Phylogenetic Diversity and Biogeography in Thalassospira Bacteria from Diverse Marine Environments
Source: PLoS One. 2014 Sep 8;9(9):e106353. doi: 10.1371/journal.pone.0106353 (PMC4157779; doi:10.1371/journal.pone.0106353)
Supplement: Figure S15 — The clustering tree of the DDH values and Radiation time of strains in the three clusters. (DOCX) [file pone.0106353.s015.docx]

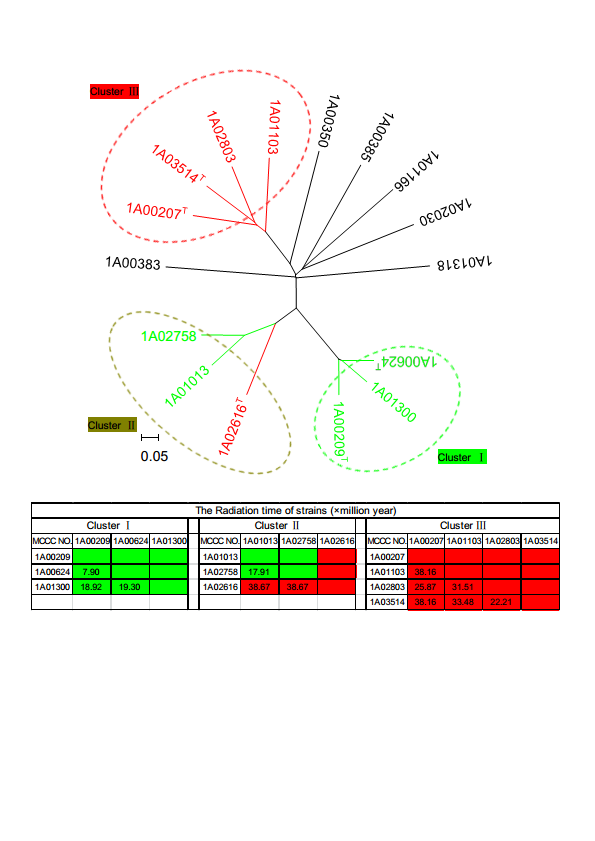


Figure S15. The clustering tree of the DDH values and Radiation time of strains in the three clusters.
