# Supplementary material for: Multilocus Sequence Analysis for Assessment of Phylogenetic Diversity and Biogeography in Thalassospira Bacteria from Diverse Marine Environments
Source: PLoS One. 2014 Sep 8;9(9):e106353. doi: 10.1371/journal.pone.0106353 (PMC4157779; doi:10.1371/journal.pone.0106353)
Supplement: Table S1 — Characteristics of the primers used in this study. (DOCX) [file pone.0106353.s016.docx]

Table S1. Characteristics of the primers used in this study

| Gene | Product name | Primer name | Sequence (5' to 3') | Size (bp) | Extension time (s) | Annealing temperature (°C) |
| --- | --- | --- | --- | --- | --- | --- |
| 16S rDNA | rRNA | 27F^†^ | AGAGTTTGATCCTGGCTCAG | 1,444 | 90 | 55.0 |
|  |  | 1492R^†^ | ACGGCTACCTTGTTACGACT |  |  |  |
|  |  | RP500^‡^ | CGTATTACCGCGGCTGCTGGCA |  |  |  |
|  |  | P300^‡^ | CCAGACTCCTACGGGAGGCAGC |  |  |  |
|  |  | PKCT^‡^ | TGCATGGCTGTCGTCAGCTCGTG |  |  |  |
| *acsA* | Acetoacetyl-CoA synthetase | acsAF^†‡^ | GGCTGGATGATGTGGAACTG | 1,000 | 60 | 46.4 |
|  |  | acsAR^†‡^ | TGGGTTTGCGAGTGCTTCCT |  |  |  |
| *aroE* | Shikimate 5-dehydrogenase | aroEF^†‡^ | TGCCGGTGTGATGGGCTGGC | 780 | 46 | 51.3 |
|  |  | aroER^†‡^ | ATCAATCCCGAACCAGCGAT |  |  |  |
| *gyrB* | DNA gyrase subunit B | gyrBF^†^ | GGCAARGARCAYTACATGGA | 1,000 | 60 | 55.0 |
|  |  | gyrBR^†^ | CGGCCCTGTTTGGCCGAWCC |  |  |  |
|  |  | gyrBRS^‡^ | CGGCCCTGTTTGGCCGA |  |  |  |
| *mutL* | DNA mismatch repair protein | mutLF^†‡^ | GATTGCCGCCGGTGAGGTGG | 810 | 50 | 46.4 |
|  |  | mutLR^†‡^ | CGCCAGAAAATCCTGATAGG |  |  |  |
| *rpoD* | RNA polymerase Sigma 70 factor | rpoDf^†^ | GAATCCCCGCTGACCATTCAMGC  GATYGTSAAYTGGCATGAC | 1,000 | 60 | 55.0 |
|  |  | rpoDr^†^ | CCAAYCGGCAAACCGACYTCGGC |  |  |  |
|  |  | rpoDfs^‡^ | GAATCCCCGCTGACCATTC |  |  |  |
| *trpB* | Tryptophan synthase beta subunit | trpBF^†‡^ | TGTCGCCGAAACCCTGATGC | 1,000 | 60 | 46.4 |
|  |  | trpBR^†‡^ | TCTGGCCGTCGTCATCCATC |  |  |  |

†: Amplification primer. ‡: Sequencing primer.
