# Supplementary material for: Multilocus Sequence Analysis for Assessment of Phylogenetic Diversity and Biogeography in Thalassospira Bacteria from Diverse Marine Environments
Source: PLoS One. 2014 Sep 8;9(9):e106353. doi: 10.1371/journal.pone.0106353 (PMC4157779; doi:10.1371/journal.pone.0106353)
Supplement: Table S2 — GenBank accession numbers of all strains used in this study. (DOCX) [file pone.0106353.s017.docx]

Table S2. GenBank accession numbers of all strains used in this study

| MCCC | 原始编号 | 16S rDNA | *rpoD* | *gyrB* | *acsA* | *aroE* | *mutL* | *trpB* |
| --- | --- | --- | --- | --- | --- | --- | --- | --- |
| MCCC 1A00207 | WP0211^T^ | **AY186195** | EU440895 | EU440894 | KJ095561 | KJ095474 | KJ095461 | KJ095354 |
| MCCC 1A00209 | M-5^T^ | **AY189753** | EU440896 | EU440840 | KJ095572 | KJ095506 | KJ095415 | KJ095379 |
| MCCC 1A00350 | R8-17 | EU440789 | EU440897 | EU440841 | KJ095526 | KJ095475 | KJ095434 | KJ095347 |
| MCCC 1A00370 | R8-8 | EU440790 | EU440898 | EU440842 | KJ095554 | KJ095511 | KJ095454 | KJ095368 |
| MCCC 1A00383 | QMT2^T^ | **AM294944*** | EU440899 | EU440893 | KJ095534 | KJ095467 | KJ095463 | KJ095401 |
| MCCC 1A00385 | R4-5 | EU440791 | EU440900 | EU440843 | KJ095551 | KJ095508 | KJ095456 | KJ095370 |
| MCCC 1A00624 | SMB34^T^ | **FJ860275** | KJ095404 | KJ095426 | KJ095573 | KJ095507 | KJ095416 | KJ095382 |
| MCCC 1A00753 | MBE#61^T^ | **AB786710** | KJ095403 | KJ095425 | KJ095577 | KJ095494 | KJ095462 | KJ095402 |
| MCCC 1A00756 | MBE#74^T^ | **AB786711** | KJ095405 | KJ095424 | KJ095576 | ND | KJ095406 | KJ095374 |
| MCCC 1A01013 | W3-1 | EU440792 | EU440901 | EU440844 | KJ095548 | KJ095482 | KJ095432 | KJ095399 |
| MCCC 1A01017 | DBT-2 | EU440793 | EU440902 | EU440845 | KJ095549 | KJ095483 | KJ095433 | KJ095400 |
| MCCC 1A01041 | PTG4-18 | EU440794 | EU440903 | EU440846 | KJ095565 | KJ095495 | KJ095417 | KJ095383 |
| MCCC 1A01051 | MARC2PI (2I) | EU440795 | EU440904 | EU440847 | KJ095570 | KJ095500 | KJ095411 | KJ095375 |
| MCCC 1A01057 | MARC4CW (3W) | EU440796 | EU440905 | EU440848 | KJ095527 | KJ095519 | KJ095435 | KJ095348 |
| MCCC 1A01072 | MARC2COD (1D) | EU440797 | EU440906 | EU440849 | KJ095563 | KJ095501 | KJ095412 | KJ095376 |
| MCCC 1A01103 | PB8B | EU440798 | EU440907 | EU440850 | KJ095556 | KJ095469 | KJ095464 | KJ095357 |
| MCCC 1A01109 | PB9B | EU440799 | EU440908 | EU440851 | KJ095552 | KJ095509 | KJ095457 | KJ095372 |
| MCCC 1A01140 | MARMC3G (5G) | EU440800 | EU440909 | EU440852 | KJ095566 | KJ095496 | KJ095418 | KJ095384 |
| MCCC 1A01148 | MARC2CO7 | EU440801 | EU440910 | EU440853 | KJ095528 | KJ095477 | KJ095436 | KJ095349 |
| MCCC 1A01166 | 35 | EU440802 | EU440911 | EU440854 | KJ095535 | KJ095516 | KJ095459 | KJ095360 |
| MCCC 1A01167 | 78 | EU440803 | EU440912 | EU440855 | KJ095536 | KJ095517 | KJ095460 | KJ095361 |
| MCCC 1A01172 | MARC2PPNC (2C) | EU440804 | EU440913 | EU440856 | KJ095550 | KJ095513 | KJ095453 | KJ095373 |
| MCCC 1A01275 | MC2-14 | EU440805 | EU440914 | EU440857 | KJ095529 | KJ095478 | KJ095437 | KJ095350 |
| MCCC 1A01288 | S31-2-1 | EU440806 | EU440915 | EU440858 | KJ095578 | KJ095520 | KJ095407 | KJ095364 |
| MCCC 1A01300 | S27-11 | EU440807 | EU440916 | EU440859 | KJ095574 | KJ095504 | KJ095422 | KJ095380 |
| MCCC 1A01318 | S25-3-2 | EU440808 | EU440917 | EU440860 | KJ095579 | KJ095521 | KJ095408 | KJ095365 |
| MCCC 1A01330 | S29-3-A | EU440809 | EU440918 | EU440861 | KJ095575 | KJ095505 | KJ095423 | KJ095381 |
| MCCC 1A01423 | S25-4 | EU440810 | EU440919 | EU440862 | KJ095580 | KJ095522 | KJ095409 | KJ095366 |
| MCCC 1A01448 | S31-7 | EU440811 | EU440920 | EU440863 | KJ095571 | KJ095502 | KJ095413 | KJ095377 |
| MCCC 1A01449 | S31-6 | EU440812 | EU440921 | EU440864 | KJ095581 | KJ095523 | KJ095410 | KJ095367 |
| MCCC 1A02030 | PR54-5 | EU440813 | EU440922 | EU440865 | KJ095524 | KJ095514 | KJ095451 | KJ095362 |
| MCCC 1A02031 | 2CR55-14 | EU440814 | EU440923 | EU440866 | KJ095525 | KJ095515 | KJ095452 | KJ095363 |
| MCCC 1A02039 | PR57-5 | EU440815 | EU440924 | EU440867 | KJ095531 | KJ095480 | KJ095430 | KJ095345 |
| MCCC 1A02040 | PR57-2 | EU440816 | EU440925 | EU440868 | KJ095532 | KJ095481 | KJ095431 | KJ095346 |
| MCCC 1A02041 | 2CR55-15 | EU440817 | EU440926 | EU440869 | KJ095557 | KJ095470 | KJ095465 | KJ095358 |
| MCCC 1A02042 | 2CR-54-5 | EU440818 | EU440927 | EU440870 | KJ095558 | KJ095471 | KJ095466 | KJ095359 |
| MCCC 1A02059 | NIC1013S-2 | EU440819 | EU440928 | EU440871 | KJ095553 | KJ095510 | KJ095458 | KJ095371 |
| MCCC 1A02060 | RC911-4 | EU440820 | EU440929 | EU440872 | KJ095555 | KJ095512 | KJ095455 | KJ095369 |
| MCCC 1A02093 | PC99-15 | EU440821 | EU440930 | EU440873 | KJ095533 | KJ095476 | KJ095439 | KJ095352 |
| MCCC 1A02094 | MC2-9 | EU440822 | EU440931 | EU440874 | KJ095567 | KJ095503 | KJ095414 | KJ095378 |
| MCCC 1A02096 | PC92-18 | EU440823 | EU440932 | EU440875 | KJ095530 | KJ095479 | KJ095438 | KJ095351 |
| MCCC 1A02616 | P-4^T^ | **EU017546** | HM365333 | HM365332 | KJ095537 | KJ095518 | KJ095443 | KJ095388 |
| MCCC 1A02753 | IB2 | EU440826 | EU440935 | EU440878 | KJ095538 | KJ095484 | KJ095440 | KJ095396 |
| MCCC 1A02758 | IB13 | EU440827 | EU440936 | EU440879 | KJ095539 | KJ095485 | KJ095444 | KJ095389 |
| MCCC 1A02767 | ID7 | EU440828 | EU440937 | EU440880 | KJ095540 | KJ095486 | KJ095445 | KJ095390 |
| MCCC 1A02785 | IH1 | EU440829 | EU440938 | EU440881 | KJ095541 | KJ095487 | KJ095446 | KJ095391 |
| MCCC 1A02803 | IK1 | EU440830 | EU440939 | EU440882 | KJ095559 | KJ095472 | KJ095428 | KJ095355 |
| MCCC 1A02843 | IP8 | EU440831 | EU440940 | EU440883 | KJ095542 | KJ095488 | KJ095447 | KJ095392 |
| MCCC 1A02866 | IU14 | EU440832 | EU440941 | EU440884 | KJ095543 | KJ095489 | KJ095448 | KJ095393 |
| MCCC 1A02873 | IV17 | EU440833 | EU440942 | EU440885 | KJ095544 | KJ095490 | KJ095449 | KJ095394 |
| MCCC 1A02878 | IX2 | EU440834 | EU440943 | EU440886 | KJ095545 | KJ095491 | KJ095441 | KJ095397 |
| MCCC 1A02898 | JB7 | EU440835 | EU440944 | EU440887 | KJ095560 | KJ095473 | KJ095429 | KJ095356 |
| MCCC 1A02921 | JG3 | EU440836 | EU440945 | EU440888 | KJ095546 | KJ095492 | KJ095442 | KJ095398 |
| MCCC 1A02935 | JK1 | EU440837 | EU440946 | EU440889 | KJ095547 | KJ095493 | KJ095450 | KJ095395 |
| MCCC 1A03005 | L6 | EU440838 | EU440947 | EU440890 | KJ095568 | KJ095497 | KJ095419 | KJ095385 |
| MCCC 1A03052 | AS-I2-11 | EU440839 | EU440948 | EU440891 | KJ095564 | KJ095498 | KJ095420 | KJ095386 |
| MCCC 1A03093 | AS-M6-11 | EU440788 | EU440949 | EU440892 | KJ095569 | KJ095499 | KJ095421 | KJ095387 |
| MCCC 1A03514 | 1-1B^T^ | **AB265822** | GU596483 | GU596484 | KJ095562 | KJ095468 | KJ095427 | KJ095353 |

Accession numbers indicated in bold were obtained from the databases. The other sequences were determined in this study.

^*^ We re-sequenced the 16S rDNA of *Thalassospira lucentensis* QMT2^T^, and showed that AM294944 is correct but not AF358664.

ND, no data. The *aroE* gene of strain MCCC 1A00756^T^ was failed to amply using several primers designed by our lab.
