# Supplementary material for: Multilocus Sequence Analysis for Assessment of Phylogenetic Diversity and Biogeography in Thalassospira Bacteria from Diverse Marine Environments
Source: PLoS One. 2014 Sep 8;9(9):e106353. doi: 10.1371/journal.pone.0106353 (PMC4157779; doi:10.1371/journal.pone.0106353)
Supplement: Table S3 — Allelic profiles of all strains used in this study. (DOCX) [file pone.0106353.s018.docx]

Table S3. Allelic profiles of all strains used in this study.

| MCCC NO. | *acsA* | *aroE* | *gyrB* | *mutL* | *rpoD* | *trpB* | STs |  | MCCC NO. | *acsA* | *aroE* | *gyrB* | *mutL* | *rpoD* | *trpB* | STs |
| --- | --- | --- | --- | --- | --- | --- | --- | --- | --- | --- | --- | --- | --- | --- | --- | --- |
| 1A00207 | 1 | 1 | 1 | 1 | 1 | 1 | 1 |  | 1A01449 | 11 | 11 | 11 | 11 | 11 | 12 | 16 |
| 1A00209 | 3 | 2 | 2 | 2 | 2 | 2 | 2 |  | 1A02030 | 12 | 12 | 12 | 12 | 12 | 13 | 17 |
| 1A00350 | 18 | 17 | 19 | 19 | 19 | 20 | 3 |  | 1A02031 | 12 | 12 | 12 | 12 | 12 | 13 | 17 |
| 1A00370 | 16 | 16 | 16 | 16 | 16 | 17 | 21 |  | 1A02039 | 13 | 13 | 13 | 13 | 13 | 14 | 18 |
| 1A00383 | 2 | 3 | 3 | 3 | 3 | 3 | 4 |  | 1A02040 | 13 | 13 | 13 | 13 | 13 | 14 | 18 |
| 1A00385 | 15 | 15 | 15 | 15 | 15 | 16 | 20 |  | 1A02041 | 14 | 14 | 14 | 14 | 14 | 15 | 19 |
| 1A00624 | 3 | 4 | 4 | 4 | 4 | 4 | 5 |  | 1A02042 | 14 | 14 | 14 | 14 | 14 | 15 | 19 |
| 1A00753 | 4 | 5 | 5 | 5 | 5 | 5 | 6 |  | 1A02059 | 15 | 15 | 15 | 15 | 15 | 16 | 20 |
| 1A00756 | 5 | 25* | 6 | 6 | 6 | 6 | 7 |  | 1A02060 | 16 | 16 | 16 | 16 | 16 | 17 | 21 |
| 1A01013 | 6 | 6 | 7 | 7 | 7 | 7 | 8 |  | 1A02093 | 17 | 17 | 17 | 17 | 17 | 18 | 22 |
| 1A01017 | 6 | 6 | 7 | 7 | 7 | 7 | 8 |  | 1A02094 | 23 | 18 | 18 | 18 | 18 | 19 | 23 |
| 1A01041 | 23 | 23 | 24 | 24 | 24 | 25 | 30 |  | 1A02096 | 18 | 19 | 19 | 19 | 19 | 20 | 24 |
| 1A01051 | 10 | 18 | 18 | 18 | 18 | 19 | 15 |  | 1A02616 | 19 | 20 | 20 | 20 | 20 | 21 | 25 |
| 1A01057 | 18 | 7 | 19 | 19 | 19 | 20 | 9 |  | 1A02753 | 21 | 22 | 22 | 22 | 22 | 23 | 27 |
| 1A01072 | 22 | 18 | 18 | 18 | 18 | 19 | 10 |  | 1A02758 | 21 | 22 | 23 | 23 | 23 | 24 | 28 |
| 1A01103 | 14 | 14 | 14 | 14 | 14 | 15 | 19 |  | 1A02767 | 21 | 22 | 23 | 23 | 23 | 24 | 28 |
| 1A01109 | 15 | 15 | 15 | 15 | 15 | 8 | 11 |  | 1A02785 | 21 | 22 | 23 | 23 | 23 | 24 | 28 |
| 1A01140 | 23 | 23 | 24 | 24 | 24 | 25 | 30 |  | 1A02803 | 20 | 21 | 21 | 21 | 21 | 22 | 26 |
| 1A01148 | 18 | 19 | 19 | 19 | 19 | 20 | 24 |  | 1A02843 | 21 | 22 | 23 | 23 | 23 | 24 | 28 |
| 1A01166 | 7 | 8 | 8 | 8 | 8 | 9 | 12 |  | 1A02866 | 21 | 22 | 23 | 23 | 23 | 24 | 28 |
| 1A01167 | 7 | 8 | 8 | 8 | 8 | 9 | 12 |  | 1A02873 | 21 | 22 | 23 | 23 | 23 | 24 | 28 |
| 1A01172 | 8 | 9 | 9 | 9 | 9 | 10 | 13 |  | 1A02878 | 21 | 22 | 22 | 22 | 22 | 23 | 27 |
| 1A01275 | 18 | 19 | 19 | 19 | 19 | 20 | 24 |  | 1A02898 | 20 | 21 | 21 | 21 | 21 | 22 | 26 |
| 1A01288 | 11 | 11 | 11 | 11 | 11 | 12 | 16 |  | 1A02921 | 21 | 22 | 22 | 22 | 22 | 23 | 27 |
| 1A01300 | 9 | 10 | 10 | 10 | 10 | 11 | 14 |  | 1A02935 | 21 | 22 | 23 | 23 | 23 | 24 | 28 |
| 1A01318 | 11 | 11 | 11 | 11 | 11 | 12 | 16 |  | 1A03005 | 23 | 23 | 24 | 24 | 24 | 25 | 30 |
| 1A01330 | 9 | 10 | 10 | 10 | 10 | 11 | 14 |  | 1A03052 | 22 | 23 | 24 | 24 | 24 | 25 | 29 |
| 1A01423 | 11 | 11 | 11 | 11 | 11 | 12 | 16 |  | 1A03093 | 23 | 23 | 24 | 24 | 24 | 25 | 30 |
| 1A01448 | 10 | 18 | 18 | 18 | 18 | 19 | 15 |  | 1A03514 | 24 | 24 | 25 | 25 | 25 | 26 | 31 |

* The *aroE* gene of strain MCCC 1A00756^T^ was failed to amply, we used Allelic type 25 here to represent it.
