# Supplementary material for: Multilocus Sequence Analysis for Assessment of Phylogenetic Diversity and Biogeography in Thalassospira Bacteria from Diverse Marine Environments
Source: PLoS One. 2014 Sep 8;9(9):e106353. doi: 10.1371/journal.pone.0106353 (PMC4157779; doi:10.1371/journal.pone.0106353)
Supplement: Table S4 — Nucleotide diversity, mearsure substitution saturation and the PHI test for single gene. (DOCX) [file pone.0106353.s019.docx]

Table S4 Nucleotide diversity, mearsure substitution saturation and the PHI test for single gene

| Locus | Nucleotide diversity | *I_SS_* | *I_SS.C_* | Results | PHI (p-value) |
| --- | --- | --- | --- | --- | --- |
| 16S rDNA | 0.012 | 0.029 | 0.783 | Little saturation | 0.001069^*^ |
| *acsA* | 0.18 | 0.215 | 0.759 | Little saturation | 0.0565 |
| *aroE* | 0.192 | 0.238 | 0.742 | Little saturation | 0.1947 |
| *gyrB* | 0.135 | 0.164 | 0.762 | Little saturation | 0.03991^*^ |
| *mutL* | 0.148 | 0.184 | 0.748 | Little saturation | 0.8193 |
| *rpoD* | 0.119 | 0.142 | 0.716 | Little saturation | 0.4708 |
| *trpB* | 0.14 | 0.1687 | 0.748 | Little saturation | 0.001069 |
