# Supplementary material for: Multilocus Sequence Analysis for Assessment of Phylogenetic Diversity and Biogeography in Thalassospira Bacteria from Diverse Marine Environments
Source: PLoS One. 2014 Sep 8;9(9):e106353. doi: 10.1371/journal.pone.0106353 (PMC4157779; doi:10.1371/journal.pone.0106353)
Supplement: Table S5 — The identity matrix of DDH and MLSA in the 16 Thalassospira bacteria. (DOCX) [file pone.0106353.s020.docx]

Table S5. The identity matrix of DDH and MLSA in the 16 *Thalassospira* bacteria

| No. | MCCC No. | Species | Group | 1 | 2 | 3 | 4 | 5 | 6 | 7 | 8 | 9 | 10 | 11 | 12 | 13 | 14 | 15 | 16 |
| --- | --- | --- | --- | --- | --- | --- | --- | --- | --- | --- | --- | --- | --- | --- | --- | --- | --- | --- | --- |
| 1 | 1A02616^T^ | *T. xianheensis* | C |  | 83.55 | 83.47 | 82.00 | 90.48 | 90.48 | 90.72 | 94.03 | 93.95 | 83.71 | 83.87 | 83.39 | 82.96 | 83.49 | 83.57 | 81.33 |
| 2 | 1A00207^T^ | *T. profundimaris* | J | 21.1 |  | 95.41 | 83.55 | 83.52 | 83.57 | 83.57 | 83.97 | 83.76 | 87.12 | 86.45 | 86.53 | 88.67 | 94.11 | 95.73 | 80.85 |
| 3 | 1A03514^T^ | *T. tepidiphila* | K | 21.3 | 62.2 |  | 83.55 | 83.01 | 83.07 | 83.28 | 83.76 | 83.68 | 87.04 | 86.40 | 86.40 | 89.25 | 94.67 | 96.16 | 81.52 |
| 4 | 1A00383^T^ | *T. lucentensis* | M | 21.6 | 22.1 | 22.4 |  | 81.92 | 81.84 | 81.79 | 81.92 | 82.08 | 83.6 | 83.23 | 83.84 | 83.15 | 83.57 | 83.57 | 79.09 |
| 5 | 1A00209^T^ | *T. xiamenensis* | A | 39.2 | 21.2 | 21.3 | 21.4 |  | **98.80** | **97.30** | 90.08 | 90.27 | 83.63 | 83.31 | 83.63 | 82.72 | 83.31 | 83.33 | 81.41 |
| 6 | 1A00624^T^ | *T. permensis* | A | 39.4 | 21.3 | 21.3 | 21.3 | **79.7** |  | **97.10** | 89.92 | 90.19 | 83.49 | 83.09 | 83.6 | 82.85 | 83.36 | 83.39 | 81.39 |
| 7 | 1A01300 | *T. xiamenensis* | A | 39.0 | 21.2 | 21.4 | 21.4 | **77.6** | **80.7** |  | 90.64 | 90.96 | 83.81 | 83.39 | 83.55 | 82.8 | 83.68 | 83.6 | 81.28 |
| 8 | 1A02758 | *Thalassospira* sp. | B | 54.2 | 21.3 | 21.4 | 21.6 | 39.6 | 39.5 | 39.1 |  | **97.30** | 83.95 | 83.87 | 83.87 | 82.69 | 83.47 | 83.79 | 81.17 |
| 9 | 1A01013 | *Thalassospira* sp. | B | 54.6 | 21.2 | 21.1 | 21.2 | 38.6 | 38.7 | 38.2 | **74.2** |  | 83.87 | 83.63 | 83.79 | 82.53 | 83.39 | 83.6 | 81.25 |
| 10 | 1A00385 | *Thalassospira* sp. | E | 21.7 | 24.9 | 24.5 | 22.7 | 21.8 | 21.7 | 21.7 | 21.8 | 21.8 |  | 89.44 | 89.12 | 86.05 | 87.04 | 87.28 | 81.49 |
| 11 | 1A01166 | *Thalassospira* sp. | F | 21.5 | 23.6 | 23.7 | 22.2 | 21.7 | 21.5 | 21.5 | 21.4 | 21.4 | 29.2 |  | 89.25 | 85.33 | 86.77 | 86.91 | 80.40 |
| 12 | 1A02030 | *Thalassospira* sp. | G | 21.4 | 23.7 | 23.8 | 22.5 | 21.6 | 21.6 | 21.8 | 21.7 | 21.4 | 63.4 | 28.3 |  | 85.97 | 86.24 | 86.59 | 81.17 |
| 13 | 1A00350 | *Thalassospira* sp. | H | 21.2 | 31.6 | 31.9 | 22.1 | 21.4 | 21.3 | 21.3 | 21.2 | 21.0 | 23.9 | 41.8 | 23.5 |  | 89.20 | 89.44 | 80.69 |
| 14 | 1A01103 | *Thalassospira* sp. | I | 21.0 | 55.3 | 56.3 | 22.5 | 21.4 | 21.2 | 21.4 | 21.6 | 21.0 | 24.4 | 23.6 | 23.8 | 31.1 |  | 94.93 | 81.01 |
| 15 | 1A02803 | *Thalassospira* sp. | L | 21.2 | 61.8 | 64.3 | 22.4 | 21.3 | 21.3 | 21.7 | 21.5 | 21.2 | 24.4 | 23.8 | 24.3 | 31.5 | 56.3 |  | 80.96 |
| 16 | 1A01318 | *Thalassospira* sp. | N | 20.2 | 20.2 | 20.6 | 20.9 | 20.6 | 20.4 | 20.8 | 21.1 | 20.3 | 20.7 | 20.1 | 20.4 | 20.9 | 23.5 | 20.7 |  |
