# Supplementary material for: Multilocus Sequence Analysis for Assessment of Phylogenetic Diversity and Biogeography in Thalassospira Bacteria from Diverse Marine Environments
Source: PLoS One. 2014 Sep 8;9(9):e106353. doi: 10.1371/journal.pone.0106353 (PMC4157779; doi:10.1371/journal.pone.0106353)
Supplement: Table S6 — The identity matrix of ANIm in the 16 Thalassospira bacteria. (DOCX) [file pone.0106353.s021.docx]

Table S6. The identity matrix of ANIm in the 16 *Thalassospira* bacteria

| No. | MCCC No. | Group | 1 | 2 | 3 | 4 | 5 | 6 | 7 | 8 | 9 | 10 | 11 | 12 | 13 | 14 | 15 | 16 |
| --- | --- | --- | --- | --- | --- | --- | --- | --- | --- | --- | --- | --- | --- | --- | --- | --- | --- | --- |
| 1 | 1A02616^T^ | C |  | 84.39 | 84.56 | 84.31 | 90.75 | 90.79 | 90.68 | 94.15 | 94.16 | 84.57 | 84.54 | 84.56 | 84.47 | 84.47 | 84.40 | 83.61 |
| 2 | 1A00207^T^ | J | 84.40 |  | 95.49 | 84.64 | 84.42 | 84.41 | 84.39 | 84.42 | 84.45 | 86.25 | 85.47 | 85.43 | 88.39 | 94.35 | 95.44 | 83.79 |
| 3 | 1A03514^T^ | K | 84.57 | 95.48 |  | 84.67 | 84.42 | 84.43 | 84.59 | 84.62 | 84.43 | 85.84 | 85.52 | 85.59 | 88.49 | 94.52 | 95.76 | 83.94 |
| 4 | 1A00383^T^ | M | 84.30 | 84.65 | 84.66 |  | 84.28 | 84.12 | 84.26 | 84.40 | 84.16 | 84.70 | 84.45 | 84.63 | 84.52 | 84.85 | 84.77 | 84.24 |
| 5 | 1A00209^T^ | A | 90.76 | 84.42 | 84.42 | 84.29 |  | **97.77** | **97.61** | 90.82 | 90.56 | 84.58 | 84.65 | 84.55 | 84.36 | 84.49 | 84.42 | 84.06 |
| 6 | 1A00624^T^ | A | 90.78 | 84.41 | 84.44 | 84.12 | **97.75** |  | **97.93** | 90.75 | 90.52 | 84.54 | 84.58 | 84.49 | 84.26 | 84.38 | 84.49 | 83.65 |
| 7 | 1A01300 | A | 90.67 | 84.39 | 84.59 | 84.27 | **97.59** | **97.92** |  | 90.66 | 90.37 | 84.57 | 84.54 | 84.86 | 84.41 | 84.54 | 84.89 | 84.23 |
| 8 | 1A02758 | B | 94.14 | 84.41 | 84.61 | 84.40 | 90.82 | 90.75 | 90.66 |  | **97.2** | 84.57 | 84.50 | 84.80 | 84.52 | 84.9 | 84.67 | 84.54 |
| 9 | 1A01013 | B | 94.15 | 84.45 | 84.43 | 84.17 | 90.55 | 90.52 | 90.38 | **97.2** |  | 84.60 | 84.56 | 84.56 | 84.48 | 84.43 | 84.51 | 83.54 |
| 10 | 1A00385 | E | 84.57 | 86.26 | 85.86 | 84.69 | 84.58 | 84.54 | 84.58 | 84.58 | 84.61 |  | 87.85 | 87.09 | 85.71 | 85.75 | 85.86 | 83.82 |
| 11 | 1A01166 | F | 84.55 | 85.47 | 85.51 | 84.45 | 84.65 | 84.58 | 84.54 | 84.51 | 84.56 | 87.85 |  | 87.14 | 85.16 | 85.50 | 85.51 | 83.34 |
| 12 | 1A02030 | G | 84.57 | 85.43 | 85.59 | 84.63 | 84.56 | 84.49 | 84.86 | 84.81 | 84.57 | 87.08 | 87.13 |  | 85.48 | 85.59 | 86.08 | 84.11 |
| 13 | 1A00350 | H | 84.49 | 88.39 | 88.48 | 84.52 | 84.37 | 84.27 | 84.42 | 84.53 | 84.50 | 85.70 | 85.16 | 85.49 |  | 88.18 | 88.33 | 84.08 |
| 14 | 1A01103 | I | 84.47 | 94.35 | 94.52 | 84.85 | 84.49 | 84.37 | 84.54 | 84.91 | 84.43 | 85.74 | 85.50 | 85.59 | 88.18 |  | 94.53 | 86.81 |
| 15 | 1A02803 | L | 84.4 | 95.44 | 95.76 | 84.76 | 84.42 | 84.49 | 84.89 | 84.67 | 84.52 | 85.85 | 85.51 | 86.08 | 88.32 | 94.53 |  | 84.09 |
| 16 | 1A01318 | N | 83.61 | 83.79 | 83.95 | 84.31 | 84.05 | 83.65 | 84.23 | 84.52 | 83.53 | 83.81 | 83.34 | 84.07 | 84.08 | 87.20 | 84.09 |  |
